# *Novosphingobium aromaticivorans* uses a Nu-class glutathione *S*-transferase as a glutathione lyase in breaking the β-aryl ether bond of lignin

**DOI:** 10.1074/jbc.RA117.001268

**Published:** 2018-02-15

**Authors:** Wayne S. Kontur, Craig A. Bingman, Charles N. Olmsted, Douglas R. Wassarman, Arne Ulbrich, Daniel L. Gall, Robert W. Smith, Larissa M. Yusko, Brian G. Fox, Daniel R. Noguera, Joshua J. Coon, Timothy J. Donohue

**Affiliations:** From the ‡ Wisconsin Energy Institute,; the § Department of Energy Great Lakes Bioenergy Research Center,; the ** Genome Center of Wisconsin, and; the Departments of ¶ Biochemistry,; ‖ Chemistry,; ‡‡ Civil and Environmental Engineering,; §§ Biomolecular Chemistry, and; ¶¶ Bacteriology, University of Wisconsin, Madison, Wisconsin 53706

**Keywords:** bacterial metabolism, enzyme mechanism, enzyme structure, lignin degradation, Escherichia coli (E. coli), beta-aryl ether, deglutathionylation, glutathione S-transferases, Novosphingobium aromaticivorans, Nu-class

## Abstract

As a major component of plant cell walls, lignin is a potential renewable source of valuable chemicals. Several sphingomonad bacteria have been identified that can break the β-aryl ether bond connecting most phenylpropanoid units of the lignin heteropolymer. Here, we tested three sphingomonads predicted to be capable of breaking the β-aryl ether bond of the dimeric aromatic compound guaiacylglycerol-β-guaiacyl ether (GGE) and found that *Novosphingobium aromaticivorans* metabolizes GGE at one of the fastest rates thus far reported. After the ether bond of racemic GGE is broken by replacement with a thioether bond involving glutathione, the glutathione moiety must be removed from the resulting two stereoisomers of the phenylpropanoid conjugate β-glutathionyl-γ-hydroxypropiovanillone (GS-HPV). We found that the Nu-class glutathione *S*-transferase NaGST_Nu_ is the only enzyme needed to remove glutathione from both (*R*)- and (*S*)-GS-HPV in *N. aromaticivorans*. We solved the crystal structure of NaGST_Nu_ and used molecular modeling to propose a mechanism for the glutathione lyase (deglutathionylation) reaction in which an enzyme-stabilized glutathione thiolate attacks the thioether bond of GS-HPV, and the reaction proceeds through an enzyme-stabilized enolate intermediate. Three residues implicated in the proposed mechanism (Thr^51^, Tyr^166^, and Tyr^224^) were found to be critical for the lyase reaction. We also found that Nu-class GSTs from *Sphingobium* sp. SYK-6 (which can also break the β-aryl ether bond) and *Escherichia coli* (which cannot break the β-aryl ether bond) can also cleave (*R*)- and (*S*)-GS-HPV, suggesting that glutathione lyase activity may be common throughout this widespread but largely uncharacterized class of glutathione *S*-transferases.

## Introduction

As society looks to diversify its sources of fuels and chemicals, there are reasons to produce them from renewable resources, such as lignocellulosic plant biomass, the most abundant organic material on Earth. Lignin, which can compose ∼25% of lignocellulosic plant biomass (1), is a heteropolymer of phenylpropanoid units linked together via several classes of covalent bonds (2). Because of its abundance, its recalcitrance to degradation, and the potential value of its aromatic substituents, there is interest in developing economical and environmentally sustainable methods to depolymerize lignin (3).

**Figure 1.**
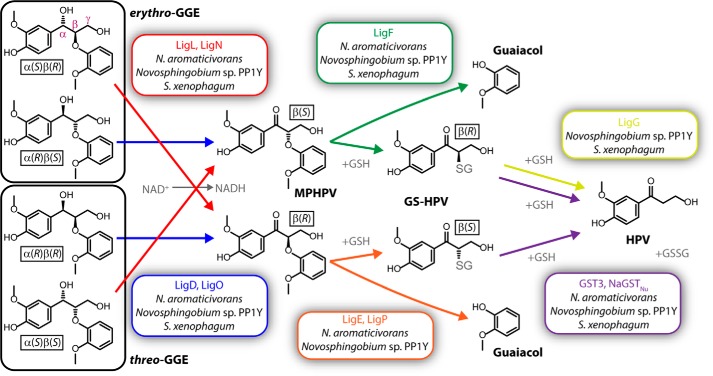
**Breaking of the β-aryl ether (β-O-4) bond of GGE via the sphingomonad β-etherase pathway.** Enzymes shown were identified in *Sphingobium* sp. SYK-6 (“Lig” enzymes (12–14)), *Novosphingobium* sp. MBES04 (GST3 (6)), or *N. aromaticivorans* DSM 12444 (NaGST_Nu_; this work). The sphingomonads investigated in this work for GGE metabolism that are predicted to contain a given enzyme (6) are listed under the enzyme name. The α-, β-, and γ-carbons are labeled in the *topmost* GGE molecule. *Erythro*-GGE consists of the α(*S*)β(*R*) and α(*R*)β(*S*) stereoisomers; *threo*-GGE consists of the α(*R*)β(*R*) and α(*S*)β(*S*) stereoisomers. All chiral molecules are labeled with their chiralities.

The β-aryl ether (β-O-4) bond typically constitutes >50% of all the linkages between aromatic units in lignin (2). Thus, methods for breaking this bond will be important for developing systems for lignin depolymerization. The β-etherase pathway, found in some sphingomonad bacteria, is a promising biological route for cleaving the β-aryl ether bond (Fig. 1). Whereas several sphingomonads, such as *Sphingobium* sp. SYK-6, *Erythrobacter* sp. SG61-1L, and *Novosphingobium* sp. MBES04, are known to contain this pathway (4–6), identifying additional species with it could advance development of biological systems for depolymerizing lignin. Ohta *et al.* (6) identified several additional sphingomonads whose genomes are predicted to contain genes for the enzymes known to be necessary for the pathway. In this work, we test three of these species, *Novosphingobium aromaticivorans* DSM 12444 (7, 8), *Novosphingobium* sp. PP1Y (9), and *Sphingobium xenophagum* NBRC 107872 (10, 11), for the ability to break the β-aryl ether bond of the dimeric aromatic compound guaiacylglycerol-β-guaiacyl ether (GGE^2^; Fig. 1) and find that *N. aromaticivorans* most rapidly and completely metabolizes GGE.

Breaking the β-aryl ether bond of GGE via the β-etherase pathway involves three steps (Fig. 1). First, the α-hydroxyl of GGE is oxidized by stereospecific NAD^+^-dependent dehydrogenases (LigL, LigN, LigD, and LigO) to generate the α-ketone, β-(2-methoxyphenoxy)-γ-hydroxypropiovanillone (MPHPV) (12). Next, stereospecific β-etherases (LigF, LigE, and LigP) replace the β-ether bond of MPHPV with a thioether bond involving glutathione (GSH), producing guaiacol and the glutathione conjugate, β-glutathionyl-γ-hydroxypropiovanillone (GS-HPV) (13–15). Finally, the glutathione moiety is removed from GS-HPV and combined with another GSH, producing hydroxypropiovanillone (HPV) and glutathione disulfide (GSSG). The Omega-class glutathione *S*-transferase (GST) LigG from *Sphingobium* sp. SYK-6 has been shown to remove the glutathione moiety from β(*R*)-GS-HPV *in vitro* (13), but no enzyme has yet been characterized from this organism that can react with the β(*S*)-stereoisomer. The Nu-class GST GST3 from *Novosphingobium* sp. MBES04 has been reported to react with both β(*R*)- and β(*S*)-GS-HPV *in vitro* (6), although the physiological role of this enzyme has not been established. Here, we find that Nu-class GSTs from *N. aromaticivorans* (NaGST_Nu_) and *Sphingobium* sp. SYK-6 (SYK6GST_Nu_) can react with both β(*R*)- and β(*S*)-GS-HPV *in vitro* and that NaGST_Nu_ is the sole enzyme required for these reactions in *N. aromaticivorans*. We also use kinetic and structural data to propose mechanisms for the glutathione lyase (deglutathionylation) reactions of NaGST_Nu_ with both GS-HPV stereoisomers.

The most distinguishing characteristic of Nu-class (Main.2 (16)) GSTs is their apparent ability to bind two molecules of GSH and/or a single molecule of GSSG (16–20). Nu-class GSTs are found in many organisms (16, 18), but their physiological roles are largely unknown. Several Nu-class GSTs have been tested for activity *in vitro*; most, including EcYfcG and EcYghU from *Escherichia coli*, showed disulfide bond reductase activity toward small molecules, such as 2-hydroxyethyl disulfide (16–18, 21), which led to the proposal that this is their main physiological activity. A strain of *E. coli* in which the *yfcG* gene was deleted was impaired in its response to oxidative stress, leading to the proposal that EcYfcG may naturally function as a peroxidase, although this enzyme exhibited low *in vitro* peroxidase activity with model peroxides (22). At least one other Nu-class GST besides GST3 has been found to have glutathione lyase activity *in vitro*: PcUre2pB1 from the white-rot fungus *Phanerochaete chrysosporium* can cleave GS-phenacylacetophenone, which led to the suggestion that this enzyme may naturally function as a glutathione lyase (19). To investigate the prevalence of glutathione lyase activity throughout the Nu class, we assayed EcYfcG and EcYghU and found that each cleaves both β(*R*)- and β(*S*)-GS-HPV *in vitro*, although less efficiently than NaGST_Nu_. Furthermore, EcYghU complements growth of an *N. aromaticivorans* ΔNaGST_Nu_ mutant, showing that it can perform these reactions *in vivo*. Our results thus suggest that glutathione lyase activity may be common throughout the Nu-class, and the lower catalytic efficiencies we find with EcYghU and EcYfcG toward GS-HPV may reflect the fact that this compound is not a natural substrate for *E. coli*.

## Results

### GGE metabolism by sphingomonads

To test for the β-etherase pathway in the sphingomonad bacteria *N. aromaticivorans* DSM 12444, *Novosphingobium* sp. PP1Y, and *S. xenophagum* NBRC 107872, we fed them *erythro*-GGE (Fig. 1), both alone and in the presence of another organic molecule, in case any of the strains can break the β-aryl ether bond of GGE but cannot use it to support growth, as is the case for *Novosphingobium* sp. MBES04 (6).

**Figure 2.**
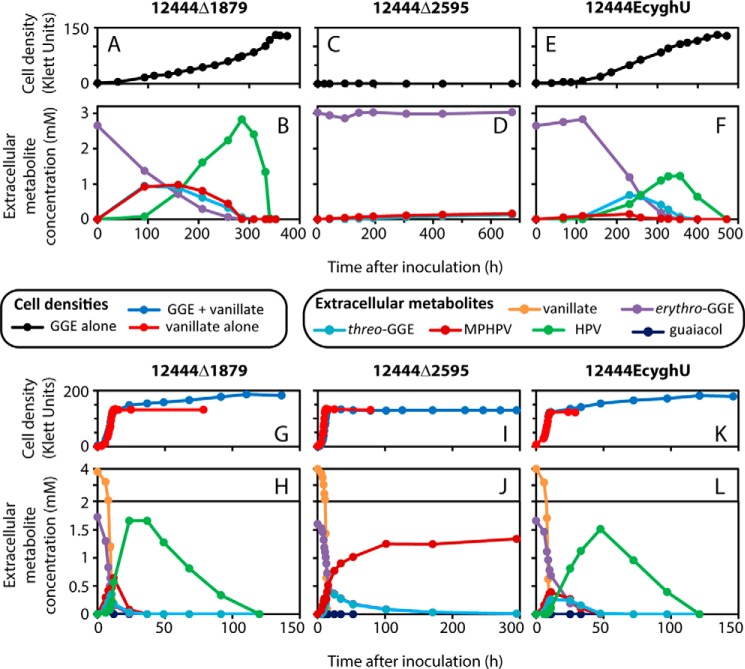
**Cell densities and extracellular metabolite concentrations of representative *N. aromaticivorans* cultures grown in SMB containing 3 mm GGE (*A–F*) or 4 mm vanillate and 1.5 mm GGE (*G–L*).** Data are shown for strains 12444Δ1879 (effective wildtype; *A*, *B*, *G*, and *H*), 12444Δ2595 (Saro_2595, which codes for NaGST_Nu_, deleted from the genome of 12444Δ1879; *C*, *D*, *I*, and *J*), and 12444EcyghU (with *E. coli yghU* replacing Saro_2595 in the genome of 12444Δ1879; *E*, *F*, *K*, and *L*). The *y*-axes of *H*, *J*, and *L* use multiple scales. For comparison, cell densities for cultures grown in SMB containing only 4 mm vanillate are included in *G*, *I*, and *K*. Cell densities are in KU; for *N. aromaticivorans*, 1 KU ∼ 8 × 10^6^ cfu/ml (Table S4).

For these studies, we created a strain of *N. aromaticivorans* in which the Saro_1879 (putative *sacB*) gene has been deleted (12444Δ1879), so that we could make additional modifications to its genome using a *sacB*-containing cloning vector. When fed *erythro*-GGE alone, *N. aromaticivorans* 12444Δ1879 completely removed the compound from the medium (Fig. 2 (*A* and *B*) and Fig. S1) and incorporated ∼22% of its organic material (measured as chemical oxygen demand (COD)) into biomass (Table S1). The majority of the electrons from the *erythro*-GGE (∼53%) were probably combined with oxygen to support respiration during growth with the compound (Table S1). When fed *erythro*-GGE plus vanillate, 12444Δ1879 completely removed both substrates from the medium (Fig. 2, *G* and *H*) and incorporated ∼41% of the COD from the vanillate (based on the results from a culture fed vanillate alone) and ∼22% of the COD from the GGE into biomass (Table S1). The known β-etherase pathway intermediates *threo-*GGE, MPHPV, and HPV transiently appeared in the media of both *erythro*-GGE–fed 12444Δ1879 cultures (Fig. 2 (*B* and *H*) and Fig. S1), whereas the pathway intermediate guaiacol was only detected at a low level in the medium of the culture fed *erythro*-GGE plus vanillate (Fig. 2*H*). The predicted pathway intermediate GS-HPV was not detected in the medium of either culture. Wild-type *N. aromaticivorans* DSM 12444 behaved similarly under both of these growth conditions, justifying our use of 12444Δ1879 as a parent strain in this study.

When fed only *erythro*-GGE, *Novosphingobium* sp. PP1Y slowly converted most of it into β-etherase pathway intermediates but did not completely remove these compounds from the medium and only achieved a low cell density (Fig. S2, *A* and *B*). When fed *erythro*-GGE plus vanillate, *Novosphingobium* sp. PP1Y rapidly consumed the vanillate, but the maximum cell density and amount of COD incorporated into biomass in this culture were the same as when the strain was fed vanillate only (Fig. S2*E* and Table S1), suggesting that the strain did not convert a significant amount of GGE into biomass in the presence of vanillate. *S. xenophagum* converted some of the *erythro*-GGE it was supplied into β-etherase pathway intermediates (Fig. S2, *D* and *H*) but did not assimilate organic material from it into cellular biomass in any culture tested: when fed *erythro*-GGE alone, no cell density was detected, and when fed *erythro*-GGE plus glucose (we found this strain to be unable to metabolize vanillate), the maximum cell density and amount of COD incorporated into biomass were the same as when the strain was fed glucose only (Fig. S2 (*C* and *G*) and Table S1). These results suggest that *N. aromaticivorans* is the best of the three organisms we analyzed for studying various aspects of the β-etherase pathway.

### Transcripts of predicted N. aromaticivorans β-etherase pathway genes increase in abundance in the presence of GGE

To test the transcriptional response of *N. aromaticivorans* to GGE, we investigated expression levels of several of its genes predicted to code for enzymes of the β-etherase pathway, including Saro_2595, which encodes a Nu-class glutathione *S*-transferase (named here NaGST_Nu_). With the exception of *ligL*, transcript levels from these genes were higher (by >6-fold) in cells grown in the presence of GGE *versus* its absence (Table 1).

**Table 1:** **Differences in transcript levels between *N. aromaticivorans* cultures grown in vanillate or vanillate plus GGE** Transcript levels for each culture were normalized to those of Saro_0141 (*rpoZ*).

Gene	BLASTp query*^a^*	Change*^b^*
		*-fold*
Saro_0205	SLG_08640, LigD (442; 78%)	11 ± 3
Saro_0793	SLG_35880, LigO (224; 41%)	6 ± 2
Saro_0794	SLG_35900, LigN (224; 45%)	9 ± 1
Saro_1875	SLG_33660, LigL (261; 49%)	1 ± 1
Saro_2091	SLG_08650, LigF (310; 59%)	8 ± 1
Saro_2405*^c^*	SLG_08660, LigE (344; 61%) SLG_32600, LigP (391; 66%)	17 ± 9
Saro_2595	MBENS4_2527, GST3 (135; 38%)	8 ± 2

*^a^* Gene product from *Sphingobium* sp. SYK-6 (SLG) or *Novosphingobium* sp. MBES04 (MBENS4) that has been shown to catalyze a reaction in the β-etherase pathway and that was used in a BLASTp search to identify the indicated *N. aromaticivorans* (Saro) gene. Bit score and percentage identity from the BLASTp searches are shown in parentheses.

*^b^* -Fold change is the ratio of the normalized transcript level in cells grown in the presence of GGE to that in cells grown in the absence of GGE for the *N. aromaticivorans* genes.

*^c^* Only Saro_2405 showed high homology (Bit score >100) to both SLG_08660 (LigE) and SLG_32600 (LigP).

### NaGST_Nu_ cleaves β(R)- and β(S)-GS-HPV

NaGST_Nu_ is 38% identical in amino acid sequence to GST3 from *Novosphingobium* sp. MBES04 (Fig. S3), which can convert β(*R*)- and β(*S*)-GS-HPV into HPV *in vitro* (Fig. 1) (6). Because *N. aromaticivorans* lacks any homologues of LigG (the enzyme from *Sphingobium* sp. SYK-6 that cleaves the β(*R*)-stereoisomer of GS-HPV (Fig. 1) (13)), we tested whether NaGST_Nu_ could cleave both β(*R*)- and β(*S*)-GS-HPV. We found that recombinant NaGST_Nu_ cleaved both stereoisomers of GS-HPV *in vitro* (Figs. S4 and S5), with slightly higher *k*_cat_ and ∼5-fold higher *K_m_* with β(*R*)-GS-HPV than with β(*S*)-GS-HPV, resulting in a ∼4-fold higher *k*_cat_/*K_m_* with the β(*S*)-isomer (Table 2).

**Table 2:** **Kinetic parameters for the enzymatic conversion of GS-HPV into HPV** Kinetic parameters are from non-linear least squares best fits to plots of initial rate ([HPV] formed/s) *versus* GS-HPV concentration using the Michaelis–Menten equation (Fig. S5).

Protein	GS-HPV*^a^*	*k*_cat_	*K_m_*	*k*_cat_/*K_m_*
		*s*^−*1*^	μ*m*	*mm*^−*1*^ *s*^−*1*^
NaGST_Nu_	β(*R*)	80 ± 10	40 ± 6	1900 ± 400
	β(*S*)	57 ± 9	8 ± 3	8000 ± 3000
NaGST_Nu_ (T51A)	β(*R*)	0.036 ± 0.005	16 ± 5	2.3 ± 0.8
	β(*S*)	0.057 ± 0.008	4 ± 2	13 ± 6
NaGST_Nu_ (Y166F)	β(*R*)	0.07 ± 0.01	110 ± 30	0.7 ± 0.2
	β(*S*)	0.16 ± 0.03	120 ± 50	1.3 ± 0.5
NaGST_Nu_ (Y224F)	β(*R*)	2 ± 1	300 ± 200	8 ± 7
	β(*S*)	1.0 ± 0.1	50 ± 9	19 ± 4
SYK6GST_Nu_	β(*R*)	13 ± 1	55 ± 7	240 ± 40
	β(*S*)	30 ± 5	11 ± 2	2700 ± 700
EcYghU	β(*R*)	0.43 ± 0.03	28 ± 4	16 ± 3
	β(*S*)	0.29 ± 0.03	12 ± 3	24 ± 6
EcYfcG	β(*R*)	0.04 ± 0.01	160 ± 60	0.2 ± 0.1
	β(*S*)	0.017 ± 0.004	130 ± 40	0.14 ± 0.06

*^a^* Stereoisomer of GS-HPV used in reaction (see Fig. 1).

### NaGST_Nu_ is necessary for GGE metabolism by N. aromaticivorans

To test for an *in vivo* role for NaGST_Nu_, we generated an *N. aromaticivorans* strain (12444Δ2595) lacking Saro_2595, the gene that encodes NaGST_Nu_. When 12444Δ2595 was provided only *erythro*-GGE, a small amount of MPHPV and *threo*-GGE appeared in the medium (Fig. 2*D*), but no cell density was detected (Fig. 2*C*), and no detectable COD was converted into biomass (Table S1). When 12444Δ2595 was fed both vanillate and *erythro*-GGE, all of the vanillate was consumed, and almost all of the GGE was converted into MPHPV (Fig. 2*J*). The maximum cell density and amount of COD incorporated into biomass were the same for this culture as for a culture fed vanillate only (Fig. 2*I* and Table S1), suggesting that 12444Δ2595 cannot convert GGE into cell material. A small amount of guaiacol appeared in the medium of the 12444Δ2595 culture fed vanillate and *erythro*-GGE (Fig. 2*J*), showing that this strain cleaved some MPHPV. However, unlike for its parent strain (12444Δ1879), no extracellular HPV was detected in any *erythro*-GGE–fed 12444Δ2595 culture (Fig. 2, *D* and *J*). These results show that NaGST_Nu_ is necessary for complete GGE metabolism by *N. aromaticivorans*.

### NaGST_Nu_ is sufficient and necessary for conversion of GS-HPV into HPV in N. aromaticivorans

**Figure 3.**
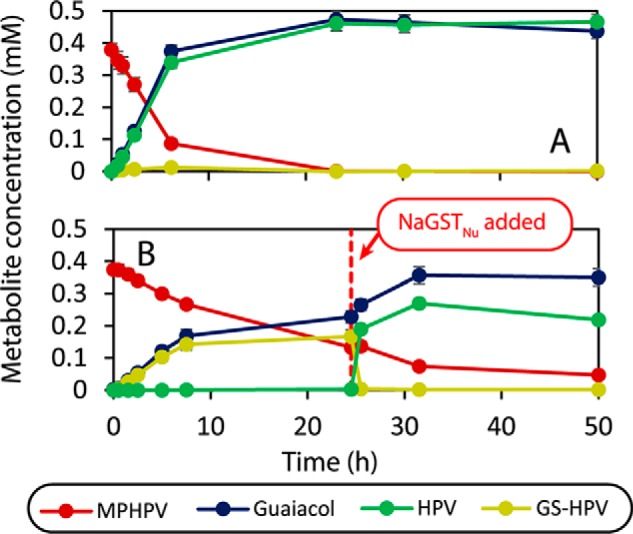
**Time courses for the reactions of *N. aromaticivorans* cell extracts with racemic β(*R*)- and β(*S*)-MPHPV.** *A*, strain 12444Δ1879. *B*, strain 12444Δ2595. The *red dotted line* in *B* indicates the time at which recombinant NaGST_Nu_ and additional GSH were added to the reaction.

To determine which step in the β-etherase pathway requires NaGST_Nu_, we incubated cell extracts of 12444Δ2595 and its parent strain (12444Δ1879) with racemic MPHPV and GSH. With the 12444Δ1879 extract, MPHPV was completely converted to roughly equimolar amounts of guaiacol and HPV, along with a small amount of GS-HPV (Fig. 3*A*). In contrast, the 12444Δ2595 extract incompletely cleaved the MPHPV, producing roughly equimolar amounts of guaiacol and GS-HPV (along with a low level of HPV, ∼2% of the level of GS-HPV formed; Fig. 3*B*). Thus, the 12444Δ2595 extract was defective in converting GS-HPV into HPV. When recombinant NaGST_Nu_ was added to the 12444Δ2595 extract after 1 day of incubation with MPHPV (Fig. 3*B*), the initially accumulated GS-HPV rapidly disappeared, with a concomitant increase in HPV, showing that the defect in GS-HPV cleavage by the 12444Δ2595 extract was caused by the lack of NaGST_Nu_.

### Structural characterization of NaGST_Nu_

**Figure 4.**
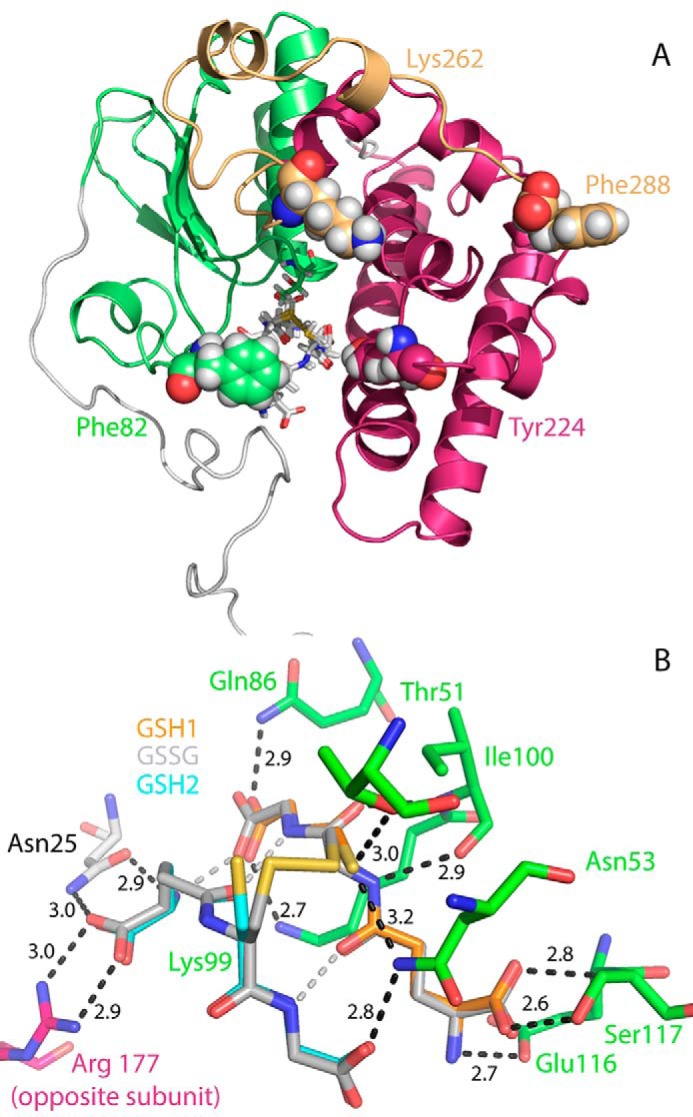
**Structure of NaGST_Nu_ (PDB 5UUO).** *A*, domain structure of one subunit of the homodimer (with the open C-terminal configuration). The monomer contains a GST1 N-terminal (thioredoxin) domain (Val^39^–Gly^129^; *green*), a GST2 C-terminal domain (Ser^135^–Leu^257^; *maroon*), and N terminus (Met^1^–Pro^38^; *white*) and C terminus (Val^258^–Phe^288^; *gold*) extensions. Atoms in Phe^82^, Tyr^224^, Lys^262^, and Phe^288^ are shown as *spheres. B*, contacts between active-site residues and the GSH1 and GSH2 dithiol (60% occupancy; *orange* and *cyan* carbon atoms, respectively) and the GS-SG disulfide (40%; *gray* carbon atoms). NaGST_Nu_ residues are *colored* according to domain origin in *A*. Interactions involving protein residues are shown in *black*; those between GSH1 and GSH2 are in *silver*. Selected distances between interacting atoms are shown.

To gain insight into the mechanism of GS-HPV cleavage by NaGST_Nu_, we solved two structures of the enzyme with resolutions of 1.25 (PDB code 5UUO) and 1.45 (PDB code 5UUN) Å (Table 3). The structures align with each other with an r.m.s. distance of 0.108 Å over 7381 atoms. NaGST_Nu_ is a homodimer; each subunit contains a characteristic N-terminal GST1 (thioredoxin-like) domain (Val^39^–Gly^129^),^3^ a C-terminal GST2 domain (Ser^135^–Leu^257^), and N-terminal (Met^1^–Pro^38^) and C-terminal (Val^258^–Phe^288^) extensions not present in most other characterized classes of GSTs (Fig. 4*A*).

**Table 3:** **Statistics for the crystal structure determinations of NaGST_Nu_** Statistics for the highest-resolution shell are shown in parentheses.

PDB entry	5UUO	5UUN
Precipitant	Ammonium sulfate	Ammonium acetate
Wavelength	0.7749	1.033
Resolution range	29.81–1.25 (1.295–1.25)	43.97–1.45 (1.502–1.45)
Space group	P 21 21 21	P 21 21 21
Unit cell	68.81, 70.39, 168.23, 90, 90, 90	68.59, 70.64, 168.57, 90, 90, 90
Total reflections	3,049,254 (309,691)	1,854,065 (113,574)
Unique reflections	225,346 (22,262)	140,421 (11,067)
Multiplicity	13.5 (13.9)	13.2 (10.3)
Completeness (%)	99.91 (99.70)	96.48 (76.88)
Mean *I*/σ(*I*)	23.67 (1.99)	36.36 (8.82)
Wilson *B*-factor	16.03	13.23
*R*_merge_	0.05235 (1.297)	0.04367 (0.2047)
*R*_meas_	0.05445 (1.346)	0.04544 (0.2159)
*R*_pim_	0.01485 (0.3565)	0.01239 (0.06599)
CC1/2	1 (0.841)	1 (0.986)
CC*	1 (0.956)	1 (0.997)
Reflections used in refinement	225,262 (22,242)	140,408 (11,066)
Reflections used for *R*_free_	1963 (197)	1852 (154)
*R*_work_	0.1303 (0.2607)	0.1357 (0.206)
*R*_free_	0.1324 (0.2979)	0.1365 (0.1595)
CC(work)	0.978 (0.913)	0.978 (0.971)
CC(free)	0.988 (0.851)	0.975 (0.9363)
Non-H atoms (total)	5658	5823
Non-H atoms (macromolecules)	4626	4567
Non-H atoms (ligands)	252	248
Non-H atoms (solvent)	780	1008
Protein residues	569	1036
r.m.s. deviation, bonds (Å)	0.007	0.007
r.m.s. deviation, angles (degrees)	1.02	1.05
Ramachandran favored/allowed/outliers (%)	93.75/2.30/0.35	97.16/2.48/0.35
Rotamer outliers (%)	0.43	0.43
Clashscore	1.89	2.13
Average *B*-factor	22.39	19.61
*B*-factor macromolecules/ligands/solvent	20.02/26.98/34.97	16.55/19.017/33.64
No. of TLS groups	9	9

Structures of Nu-class GSTs solved in the presence of GSH typically contain either two GSH molecules (*e.g.* PDB codes 3C8E and 4IKH) or a single GSSG molecule (*e.g.* PDB codes 4MZW and 3GX0) in each active site. Consistent with this, each NaGST_Nu_ subunit contained electron density data that were best modeled as a mixed population of GSH1 and GSH2 thiols with an HS–SH distance of 2.4 Å (∼60% occupancy) and a GSSG disulfide with S–S distance of 2.0 Å (∼40% occupancy) (Fig. 4*B* and Fig. S6), suggesting a heterogeneous population of protein molecules in the crystals in which each subunit contained either two GSHs or a single GSSG (see legend to Fig. S6).

In NaGST_Nu_, seven residues make close contacts with GSH1 (Thr^51^, Asn^53^, Gln^86^, Lys^99^, Ile^100^, Glu^116^, and Ser^117^), and three residues make close contacts with GSH2 (Asn^25^, Asn^53^, and Arg^177^ from the opposite chain in the dimer) (Fig. 4*B*). These residues and contacts with the GSHs are conserved throughout much of the Nu class (Fig. S3) (16, 19, 20), although members of this family with truncated N termini lack an analogue of Asn^25^ (Fig. S3; see supplemental materials of Ref. 19).

**Figure 5.**
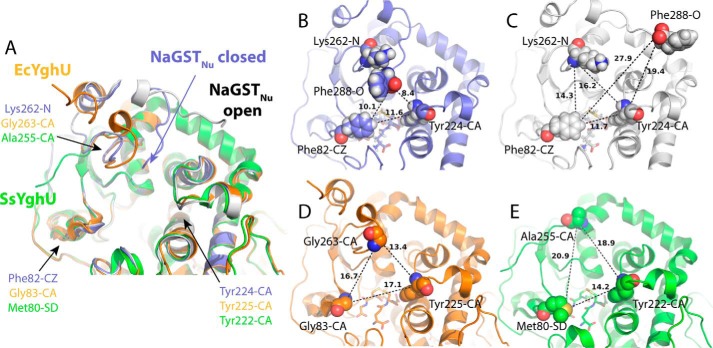
**Comparison of the region surrounding the active site in closely related Nu-class GSTs.** *A*, alignment of subunits of NaGST_Nu_ (PDB code 5UUO) (*blue*, closed C-terminal configuration; *white*, open C-terminal configuration), EcYghU (PDB code 3C8E; *orange*), and SsYghU (PDB code 4MZW; *green*). Each subunit is labeled at its C terminus. Residues labeled in *B–E* with atoms shown as *spheres* are spatially conserved between the subunits and define a triangle used to approximate the area of each active-site channel opening. *B*, closed configuration of NaGST_Nu_ (channel opening ∼11 Å^2^). *C*, open configuration of NaGST_Nu_ (channel opening ∼18 Å^2^). *D*, EcYghU (channel opening ∼25 Å^2^). *E*, SsYghU (channel opening ∼25 Å^2^).

In NaGST_Nu_, a short channel connects the active site to the solvent (Fig. 5 and Fig. S7). This channel is also present in Nu-class GSTs from *E. coli* (EcYghU; PDB code 3C8E (18)) and *Streptococcus sanguinis* SK36 (SsYghU; PDB code 4MZW). In most other structurally characterized Nu-class GSTs, the active site is more solvent-exposed, because these proteins lack N-terminal residues that contribute to one of the channel walls (Fig. S3; see supplemental materials of Ref. 19).

In each NaGST_Nu_ homodimer we solved (PDB codes 5UUN and 5UUO), the individual monomers differ in the positioning of residues Gln^282^–Phe^288^ (Fig. 5, *A–C*). The C-terminal Phe^288^ resides near the active-site channel in one subunit (NaGST_Nu_ closed; Fig. 5*B*) and ∼18 Å away from the channel entrance in the other (NaGST_Nu_ open; Fig. 5*C*), resulting in a difference between the monomers in the opening to the active-site pocket. The closed NaGST_Nu_ configuration is stabilized by hydrogen bonds between Lys^286^ and both Lys^262^ and Arg^220^. The open configuration lacks these interactions, and its Arg^220^ side chain has two rotamer positions. In other structurally characterized Nu-class GST homodimers, the individual subunits are symmetric, with C termini that generally extend away from the active site (alignments for EcYghU and SsYghU subunits are shown in Fig. 5 (*A*, *D*, and *E*)).

### Modeling of substrate binding and proposed reaction mechanism

**Figure 6.**
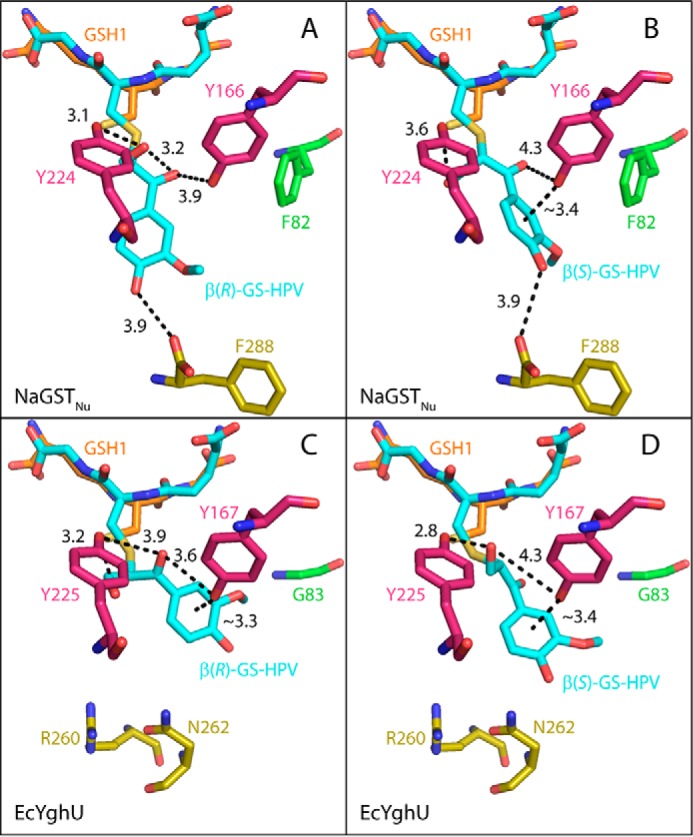
**Modeling of β(*R*)- and β(*S*)-GS-HPV into the NaGST_Nu_ and EcYghU active sites.** The glutathione moiety of GS-HPV is modeled into the position occupied by GSH2 in the structures. Carbon atoms of GS-HPV are *cyan*. Those of GSH1 are *orange. Coloring* of NaGST_Nu_ residues is the same as in Fig. 4. EcYghU residues are *colored* the same as their NaGST_Nu_ analogues in Fig. 4. *A* and *B* show modeling into NaGST_Nu_ (PDB code 5UUO; closed C-terminal configuration). *C* and *D* show modeling into EcYghU (PDB code 3C8E). *A* and *C* show predicted interactions involving β(*R*)-GS-HPV. *B* and *D* show predicted interactions involving β(*S*)-GS-HPV. Residues Phe^82^ and Phe^288^ in NaGST_Nu_ and Arg^260^ and Asn^262^ in EcYghU contribute to different internal dimensions of the active-site channels of NaGST_Nu_ and EcYghU, leading to different predicted orientations of the bound substrates between the enzymes. Fig. S7 shows *space-filling models* of the enzyme-substrate complexes.

We separately modeled the GS- moiety of β(*R*)- and β(*S*)-GS-HPV into the GSH2 active-site position (Fig. 4*B*) of the closed C-terminal configuration subunit of NaGST_Nu_ and found that the HPV moieties extend into the active-site channel in different orientations without generating unfavorable steric clashes (Fig. 6 (*A* and *B*) and Fig. S7 (*A* and *B*)). For both GS-HPV stereoisomers, there are predicted hydrogen bonds between the γ-hydroxyl and the hydroxyl of Tyr^224^ and between the HPV phenolic group and the carboxyl group of the C-terminal Phe^288^ (Fig. 6, *A* and *B*). For β(*R*)-GS-HPV, the γ-hydroxyl is also predicted to hydrogen-bond with the α-ketone, which in turn is predicted to hydrogen-bond with the hydroxyl of Tyr^166^ (Fig. 6*A*). For β(*S*)-GS-HPV, the hydroxyl of Tyr^166^ is predicted to form a hydrogen bond with the HPV aromatic ring (24) and a long-range interaction with the HPV α-ketone (Fig. 6*B*).

**Figure 7.**
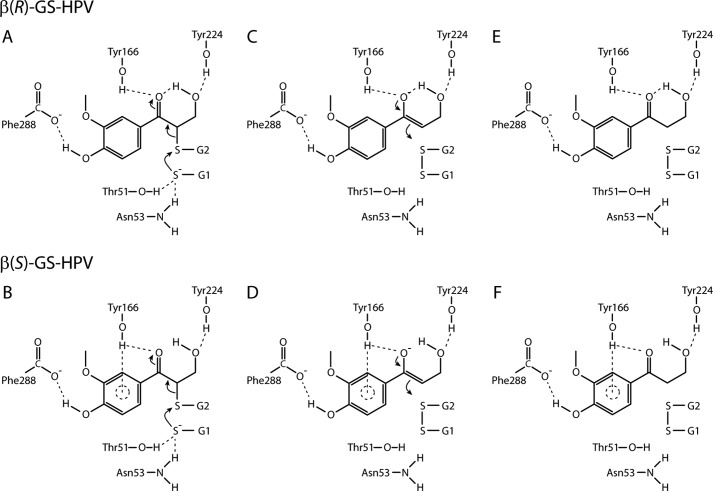
**Proposed mechanism for NaGST_Nu_-catalyzed cleavage of the thioether bond in β(*R*)-GS-HPV (*top row*) or β(*S*)-GS-HPV (*bottom row*).** *A* and *B*, Thr^51^ and Asn^53^ provide hydrogen bonds that stabilize a reactive GS1 thiolate anion, which attacks the GS- moiety of GS-HPV (occupying the active-site GSH2 position) to form a G1S-SG2 disulfide. *C* and *D*, rupture of the thioether bond is facilitated by formation of a transient enolate intermediate, which is stabilized by interactions between GS-HPV and Tyr^166^ and Tyr^224^ of NaGST_Nu_. *E* and *F*, capture of a solvent-derived proton by the carbanion collapses the enolate to form HPV. The hydroxyl group of the C-terminal Phe^288^ in the closed NaGST_Nu_ configuration provides a hydrogen bond that stabilizes the positioning of the substrate throughout the process.

We propose that the thiol of a GSH molecule in the GSH1 active-site position of NaGST_Nu_ is activated by hydrogen-bonding with the side-chain hydroxyl of Thr^51^ (3.0 Å) and the side-chain amide of Asn^53^ (3.2 Å) (Figs. 4*B* and 7 (*A* and *B*)). These residues are part of a Thr-Pro-Asn motif that is highly conserved throughout the Nu class, the Asn residue of which was found to be critical for the glutathione lyase activity of PcUre2pB1 (19). Interactions with active-site residues have been shown to lower the p*K_a_* of a GSH thiol and stabilize the reactive thiolate anion in members of other GST classes (*e.g.* the GSH thiol p*K_a_* is lowered to ∼6.6 by hydrogen bonds with Tyr and Arg side chains in human GSTA3-3 (25)). In addition, the Thr^51^ hydroxyl closely contacts the backbone amides of Asn^53^ and Gly^54^, and the side-chain amide of Asn^53^ closely contacts the side-chain amine of Lys^56^ and the Gly carboxylate of the GS- moiety of GS-HPV (Fig. S8). Extended hydrogen-bonding networks (“second-sphere interactions”) such as this are proposed to contribute to GSH thiolate stabilization in other GST classes (26).

In our proposed reaction mechanism for NaGST_Nu_, the GS1 thiolate attacks the thioether of GS-HPV to form a disulfide GS-SG. Because the C–S bond lyase reaction proceeds in the absence of a redox cofactor, such as flavin or pyridine nucleotide, we propose that a transient enolate (Fig. 7, *C* and *D*) stores the 2e^−^ reducing equivalents released by disulfide bond formation. Formation of this enolate is proposed to be facilitated by polarization of the GS-HPV α-ketone, which is stabilized by the predicted interactions between GS-HPV and Tyr^166^ and Tyr^224^ (Figs. 6 and 7 (*C* and *D*)). Due to active-site steric constraints, our modeling places the reactive portion (S-C_β_-(C_α_=O)-aryl) of both β(*R*)- and β(*S*)-GS-HPV into roughly planar configurations in the NaGST_Nu_ channel (Fig. 6 (*A* and *B*) and Fig. S7 (*A* and *B*)), which should also promote enolate formation. Collapse of the proposed enolate intermediate proceeds with carbanion trapping of a solvent-derived proton, corresponding to reduction of the carbon atom originally containing the thioether bond (Fig. 7, *E* and *F*).

This proposed mechanism for NaGST_Nu_, in which the activated thiol of a glutathione molecule is used to break the thioether bond of GS-HPV, is different from that proposed for the Omega-class GST LigG, in which the activated thiol of a Cys residue in the enzyme breaks the thioether bond (27). In contrast to NaGST_Nu_, LigG is not known or predicted to be able to bind two glutathione molecules in a single active site (27, 28). NaGST_Nu_ contains a single Cys residue (Fig. S3), whose thiol is >20 Å away from either GSH thiol, and so is unlikely to be involved in the lyase reaction.

### Single-amino acid variants of NaGST_Nu_ are catalytically impaired

To test the plausibility of our proposed reaction mechanism, three residues in NaGST_Nu_ predicted to be important for catalysis were separately mutated: Thr^51^ into Ala (T51A), Tyr^166^ into Phe (Y166F), and Tyr^224^ into Phe (Y224F). All three mutated enzymes had decreased reactivity with GS-HPV compared with wildtype NaGST_Nu_ (Fig. S5 (*B–D*) and Table 2). The T51A variant had *K_m_* values similar to wildtype NaGST_Nu_ but *k*_cat_ values that were ∼1000-fold lower, consistent with our proposal that Thr^51^ is primarily involved in stabilizing the reactive GSH1 thiolate and not in binding GS-HPV. Compared with wildtype NaGST_Nu_, the Y166F variant had *k*_cat_ values that were ∼1000- and ∼400-fold lower and *K_m_* values that were ∼2.5- and ∼15-fold higher for the β(*R*)- and β(*S*)-stereoisomers of GS-HPV, respectively. These results suggest that the Tyr^166^ hydroxyl is critical for catalysis with both GS-HPV stereoisomers and is important for binding of β(*S*)-GS-HPV. Compared with wildtype NaGST_Nu_, the Y224F variant had *k*_cat_ values that were ∼40- and ∼60-fold lower and *K_m_* values that were ∼7.5- and ∼6-fold higher for the β(*R*)- and β(*S*)-stereoisomers of GS-HPV, respectively. These results implicate the Tyr^224^ hydroxyl in both binding and catalysis, although it is less important to catalysis than that of Tyr^166^.

### Modeling GS-conjugated syringyl phenylpropanoids into the NaGST_Nu_ active site

Besides GS-HPV, depolymerization of natural lignin oligomers via the β-etherase pathway is expected to produce syringyl phenylpropanoids (3). Modeling of the β(*R*)- and β(*S*)-isomers of a GS-conjugated syringyl phenylpropanoid into the NaGST_Nu_ active site predicts that they should bind in essentially the same orientations as the corresponding GS-HPV isomers, because the active-site channel can accommodate each syringyl conjugate's additional methoxy group (Fig. S7, *C* and *D*). Thus, Nu-class GSTs may be the only enzymes necessary for the glutathione lyase step in deconstructing natural lignin oligomers via the β-etherase pathway.

### A Nu-class GST from Sphingobium sp. SYK-6 can cleave β(R)- and β(S)-GS-HPV

Although the sphingomonad *Sphingobium* sp. SYK-6 can metabolize GGE (5, 12), and its LigG enzyme can cleave β(*R*)-GS-HPV (13), no enzyme capable of cleaving β(*S*)-GS-HPV has been identified in this organism (Fig. 1). To identify an enzyme capable of cleaving β(*S*)-GS-HPV, we tested a Nu-class GST from *Sphingobium* sp. SYK-6 (with 63% sequence identity to NaGST_Nu_; Fig. S3) encoded by SLG_04120 and named here SYK6GST_Nu_ and found that recombinant SYK6GST_Nu_ cleaved both β(*S*)- and β(*R*)-GS-HPV *in vitro* (Fig. S5*E*). SYK6GST_Nu_ had higher *k*_cat_ and lower *K_m_* with β(*S*)-GS-HPV than with β(*R*)-GS-HPV, leading to a ∼10-fold greater *k*_cat_/*K_m_* with the β(*S*)-isomer (Table 2). Thus, SYK6GST_Nu_ could cleave β(*S*)-GS-HPV and potentially contribute, along with LigG, to β(*R*)-GS-HPV cleavage in *Sphingobium* sp. SYK-6 (13).

### Nu-class GSTs from E. coli can cleave β(R)- and β(S)-GS-HPV

To investigate the prevalence of glutathione lyase activity throughout the GST Nu-class, we tested two Nu-class GSTs from *E. coli* (EcYghU and EcYfcG), an organism not known to metabolize lignin-derived glutathione conjugates. We found that recombinant EcYghU cleaves β(*R*)- and β(*S*)-GS-HPV *in vitro* (Fig. S5*F*); whereas its *K_m_* values were comparable with those of NaGST_Nu_, its *k*_cat_ values were much lower, resulting in *k*_cat_/*K_m_* values for EcYghU ∼100-fold lower than those of NaGST_Nu_ (Table 2). To investigate the cause of these differences in catalytic efficiency, we modeled β(*R*)- and β(*S*)-GS-HPV into the active site of EcYghU (PDB code 3C8E). Whereas EcYghU is similar to NaGST_Nu_ in sequence (61% identical, including analogues of catalytic residues Thr^51^, Tyr^166^, and Tyr^224^; Fig. S3) and structure (r.m.s. deviation of 0.49 Å over 3116 atoms; Fig. 5), there are steric differences in the interior of the active-site channel that lead to different predicted orientations of the bound substrates between the enzymes (Fig. 6 and Fig. S7). The hydroxyls of Tyr^167^ and Tyr^225^ in EcYghU (analogues of Tyr^166^ and Tyr^224^ in NaGST_Nu_) are predicted to interact with the HPV moiety of both β(*R*)- and β(*S*)-GS-HPV (Fig. 6, *C* and *D*). For β(*R*)-GS-HPV, Tyr^225^ is predicted to form hydrogen bonds with both the γ-hydroxyl and α-ketone, and Tyr^167^ is predicted to form hydrogen bonds with the α-ketone and the aromatic ring (Fig. 6*C*). For β(*S*)-GS-HPV, Tyr^225^ is predicted to form a hydrogen bond with the γ-hydroxyl, and Tyr^167^ is predicted to form a hydrogen bond with the aromatic ring and a long-range interaction with the γ-hydroxyl (Fig. 6*D*). These interactions could help stabilize an enolate intermediate in the reactions between EcYghU and GS-HPV, so EcYghU could follow a reaction mechanism similar to that proposed for NaGST_Nu_ (Fig. 7). However, the reactive atoms (S-C_β_-(C_α_=O)-aryl) of both β(*R*)- and β(*S*)-GS-HPV bound to EcYghU are predicted to be ∼45° out of alignment (Fig. 6 (*C* and *D*) and Fig. S7 (*E* and *F*)), which could hinder formation of the enolate and lead to the lower *k*_cat_ values for EcYghU than for NaGST_Nu_ (for which these reactive atoms were in a roughly planar configuration (Fig. 6 (*A* and *B*) and Fig. S7 (*A* and *B*)).

Despite these lower *in vitro k*_cat_ values, we found that EcYghU was able to substitute for NaGST_Nu_ in the β-etherase pathway *in vivo*: a strain of *N. aromaticivorans* (12444EcyghU) in which Saro_2595 was replaced in the genome by the *E. coli yghU* gene completely removed the GGE from the media of both of its cultures and assimilated the GGE into biomass (Fig. 2 (*E*, *F*, *K*, and *L*) and Table S1), whereas 12444Δ2595 (which lacks NaGST_Nu_) could not (Fig. 2, *C*, *D*, *I*, and *J*). 12444EcyghU metabolized GGE slower than 12444Δ1879 (which contains NaGST_Nu_) (Fig. 2), which we propose reflects the slower conversion of GS-HPV into HPV in 12444EcyghU than in 12444Δ1879, due to the lower catalytic efficiencies of EcYghU than NaGST_Nu_.

The other *E. coli* Nu-class GST we tested, EcYfcG, is less similar to NaGST_Nu_ in sequence (42% identical; Figs. S3 and S9) and structure (PDB code 3GX0; r.m.s. deviation of 0.775 Å over 2016 atoms and lacking an enclosed active-site channel (17)). Whereas we found that recombinant EcYfcG cleaved both β(*R*)- and β(*S*)-GS-HPV *in vitro* (Fig. S5*G*), *K_m_* values were higher and *k*_cat_ values were much lower than those of NaGST_Nu_, leading to *k*_cat_/*K_m_* values with GS-HPV ∼10,000-fold lower for EcYfcG than for NaGST_Nu_ (Table 2). The lower catalytic efficiencies of EcYfcG are probably due, at least in part, to the absence of analogues of NaGST_Nu_ residues Tyr^166^ and Tyr^224^ (Fig. S3), which were found to be important for catalysis and substrate binding in NaGST_Nu_. Given the dramatically lower catalytic efficiencies of EcYfcG, it is unlikely that EcYfcG could substitute for NaGST_Nu_ in *N. aromaticivorans*.

## Discussion

In developing bio-based systems to generate products from renewable materials such as lignin, optimized cellular and enzyme catalysts are needed. Toward this end, we tested sphingomonads for the ability to break the β-aryl ether bond commonly found in lignin (Fig. 1) and identified and characterized a Nu-class glutathione *S*-transferase from *N. aromaticivorans* that acts as a glutathione lyase in the process (NaGST_Nu_).

### Differences in β-aryl ether bond breaking by sphingomonads

We found that *N. aromaticivorans* was the most effective species studied here at breaking the β-aryl ether bond of the dimeric aromatic compound GGE and assimilating GGE into cellular biomass. The rate of GGE β-aryl ether bond breaking by *N. aromaticivorans* (∼165 μm in ∼25 h; Fig. S1) is comparable with rates for *Erythrobacter* sp. SG61-1L (∼180 μm in ∼74 h (5)) and *Novosphingobium* sp. MBES04 (∼700 μm in ∼40 h (6)). *Sphingobium* sp. SYK-6 (∼180 μm in ∼160 h (5)), *Novosphingobium* sp. PP1Y (Fig. S2), and *S. xenophagum* (Fig. S2) are slower and/or less efficient at breaking the β-aryl ether bond of GGE under laboratory conditions, although they each contain enzymes implicated in the β-etherase pathway. Understanding the bases for these differences can aid future efforts to develop microbial systems for converting lignocellulosic biomass into commodities.

### GGE metabolism by N. aromaticivorans

The transient appearance of extracellular MPHPV, *threo*-GGE, and HPV in *N. aromaticivorans* cultures fed *erythro*-GGE suggests that the bacterium excreted these metabolites and then subsequently transported them back into the cell (Fig. 2). The production of *threo*-GGE in cultures fed *erythro*-GGE suggests that MPHPV reduction occurs *in vivo*, as was previously found for *Pseudomonas acidovorans* D3 (29), because the two forms of GGE are not directly interconvertible. The low level of extracellular guaiacol and the absence of extracellular GS-HPV suggest that MPHPV cleavage occurred intracellularly, as was proposed for *Novosphingobium* sp. MBES04 (6), and that the products of this cleavage were largely retained within the cells. Because we did not observe any additional aromatic compounds in our culture media, any aromatic metabolites downstream of HPV and guaiacol were also probably largely retained within the cells.

Because NaGST_Nu_ catalyzes the conversion of GS-HPV into HPV, the accumulation of extracellular MPHPV by *N. aromaticivorans* 12444Δ2595 fed *erythro*-GGE and vanillate (Fig. 2*J*) was unexpected. Cleavage of MPHPV into guaiacol and GS-HPV is catalyzed by LigF and LigE (Fig. 1), enzymes that are probably expressed in 12444Δ2595, because crude extract from this strain can cleave MPHPV (Fig. 3*B*). We hypothesize that without NaGST_Nu_, GS-HPV accumulates in 12444Δ2595, and cells become limited for the free GSH that is needed to cleave MPHPV.

It is unclear whether the trace amount of HPV formed in assays using 12444Δ2595 extract (Fig. 3*B*) resulted from activity of an unknown enzyme or spontaneous cleavage of GS-HPV. Even if there were another enzyme in *N. aromaticivorans* with low glutathione lyase activity, our results with the 12444Δ2595 strain and its extract show that NaGST_Nu_ is sufficient for cleavage of GS-HPV and necessary for complete metabolism of GGE by *N. aromaticivorans*.

### The role of Nu-class GSTs in the β-etherase pathway

This work demonstrates that a Nu-class GST is probably the only enzyme necessary for metabolizing the β(*R*)- and β(*S*)-isomers of lignin-derived GS-phenylpropanoids (both guaiacyl (HPV) and syringyl) in nature. This raises the question of why some sphingomonads (such as *Sphingobium* sp. SYK-6) contain both a Nu-class GST and LigG, an Omega-class GST that is reported to be specific for the β(*R*)-isomer (Fig. 1) (13). Indeed, cell extract from a *Sphingobium* sp. SYK-6 ΔLigG mutant was found to completely cleave racemic GS-HPV, presumably because the lysate contained active SYK6GST_Nu_ (13). Notably, we found that the *k*_cat_/*K_m_* value for SYK6GST_Nu_ with β(*R*)-GS-HPV (∼240 mm^−1^ s^−1^; Table 2) is ∼7-fold lower than that reported for LigG with β(*R*)-GS-HPV (∼1700 mm^−1^ s^−1^ (28)). Thus, whereas SYK6GST_Nu_ probably cleaves β(*S*)-GS-HPV in *Sphingobium* sp. SYK-6, LigG may play an important role in cleaving β(*R*)-GS-HPV in that organism.

*Novosphingobium* sp. MBES04 contains not only a LigG homologue (GST6, which preferentially reacts with β(*R*)-GS-HPV (6)), but also two Nu-class GSTs (GST3 and a protein encoded by MBENS4_4395). Whereas GST3 cleaves β(*R*)- and β(*S*)-GS-HPV *in vitro* (6), its catalytic efficiencies and physiological role have not been reported. No investigation of MBENS4_4395 has been reported, but it is 66% identical to NaGST_Nu_ in amino acid sequence and contains analogues of catalytic residues Tyr^166^ and Tyr^224^ (Fig. S3) and so can probably react with β(*R*)- and β(*S*)-GS-HPV. Whereas the relative roles of these enzymes in *Novosphingobium* sp. MBES04 are unknown, given the effect of deleting NaGST_Nu_ on *N. aromaticivorans* (its only known glutathione lyase) (Fig. 2, *C*, *D*, *I*, and *J*), perhaps they represent redundant enzymes or are specialized for different GS-conjugates.

### The potential roles of Nu-class GSTs as glutathione lyases in organisms that do not contain the β-etherase pathway

All five of the Nu-class GSTs that have been tested for GS-HPV cleavage (NaGST_Nu_, SYK6GST_Nu_, EcYghU, and EcYfcG (tested here), and GST3 (6)) show *in vitro* glutathione lyase (deglutathionylation) activity with the β(*R*)- and β(*S*)-stereoisomers of this substrate, although with a wide range of catalytic efficiencies. Phylogenetic analysis of Nu-class GSTs shows that these five enzymes lie in separate subclades (Fig. S9), although the residues predicted to be involved in the binding of two GSH molecules (or one GSH molecule and the glutathione moiety of a GS-conjugate) (Fig. 4*B*) and in activating the thiol of the GSH1 molecule (Thr^51^ and Asn^53^) are conserved in many members of the class (Fig. S3) (16, 19, 20). Another Nu-class GST, PcUre2pB1 from *P. chrysosporium*, has also been shown to exhibit *in vitro* glutathione lyase activity (19). Glutathione lyase activity may thus be widespread throughout this large class of GSTs, most of which are found in organisms (like *E. coli*) not known or predicted to break the β-aryl ether bond of lignin. Whereas EcYghU and EcYfcG cleave β(*R*)- and β(*S*)-GS-HPV *in vitro* with lower catalytic efficiencies than NaGST_Nu_ and SYK6GST_Nu_ (Table 2), this may reflect the fact that GS-HPV is not a natural substrate for the *E. coli* enzymes. Although the overall structures of Nu-class GSTs are similar, differences between them, particularly in the region surrounding the active site where the GS-conjugate would be located, could make individual enzymes optimally suited for binding and cleaving GS-conjugates that their respective organisms encounter more frequently. For example, we propose that steric differences in the active-site channels between NaGST_Nu_ and EcYghU contribute to the observed differences in catalytic efficiencies between these enzymes with GS-HPV as substrate (Fig. 6 and Fig. S7).

### Conclusions

The rate of GGE metabolism by *N. aromaticivorans* makes it an attractive organism for studying various aspects of the β-etherase pathway and may allow it to be developed into a biological system for converting lignin oligomers into useful compounds. Our finding that NaGST_Nu_ is the only enzyme needed for the glutathione lyase step of the β-etherase pathway is notable, because the other pathway steps require multiple stereospecific enzymes (Fig. 1). In addition, this is the first demonstration of a Nu-class GST having a defined physiological role as a glutathione lyase in an organism. Our analyses of wild-type NaGST_Nu_ and its variants define roles for residues conserved throughout the Nu class in GS-HPV cleavage. Our identification of SYK6GST_Nu_ probably solves the enigma of how both β(*R*)- and β(*S*)-GS-HPV are cleaved in *Sphingobium* sp. SYK-6: based on kinetic analyses, we propose that SYK6GST_Nu_ cleaves β(*S*)-GS-HPV, whereas LigG and SYK6GST_Nu_ both contribute to cleaving β(*R*)-GS-HPV in that organism. Finally, the ability of EcYghU and EcYfcG to cleave GS-HPV shows that Nu-class GSTs from organisms lacking the β-etherase pathway can also act as glutathione lyases, offering new possibilities for the physiological roles of members of this large enzyme class.

## Experimental procedures

### Bacterial strains and growth media

Strains used are listed in Table S2. We deleted Saro_1879 (putative *sacB*; SARO_RS09410 in the recently reannotated genome in NCBI) from the *N. aromaticivorans* DSM 12444 genome to create a strain (12444Δ1879) amenable to markerless genomic modifications using a variant of pK18*mobsacB* (30), which contains *sacB* and a kanamycin resistance gene. We used 12444Δ1879 as parent strain to generate strains in which Saro_2595 (SARO_RS13080 in the recently reannotated genome in NCBI) was deleted from the genome (12444Δ2595) and in which Saro_2595 was replaced in the genome by the *E. coli* DH5α *yghU* gene (12444EcyghU). Detailed methods for modifying the genome of *N. aromaticivorans* are described in the supporting information. All plasmids and primers used for genomic modifications are contained in Tables S2 and S3. Fig. S10 shows genotypes for 12444Δ2595 and 12444EcyghU.

Unless otherwise noted, *E. coli* cultures were grown in lysogeny broth and shaken at ∼200 rpm at 37 °C. For routine storage and manipulation, sphingomonad cultures were grown in lysogeny broth or GluSis at 30 °C. GluSis is a modification of Sistrom's minimal medium (31) in which the succinate has been replaced by 22.6 mm glucose. Standard mineral base (SMB) (32) used for growth experiments contains 20 mm Na_2_HPO_4_, 20 mm KH_2_PO_4_, 1 g/liter (NH_4_)_2_SO_4_, and 20 ml of Hutner's vitamin-free concentrated base (adapted from Ref. 33 but lacking nicotinic acid, thiamin, and biotin; see supporting information) per liter, final pH 6.8. SMB was supplemented with carbon sources as described below. Where needed to select for plasmids, media were supplemented with 50 μg/ml kanamycin and/or 20 μg/ml chloramphenicol.

### Sphingomonad growth experiments

Cell densities were measured using a Klett–Summerson photoelectric colorimeter with a red filter. For *N. aromaticivorans*, 1 Klett unit (KU) is equal to ∼8 × 10^6^ cfu/ml (Table S4). Experimental cultures of *N. aromaticivorans* and *Novosphingobium* sp. PP1Y were grown in SMB containing either vanillate or GGE alone (4 and 3 mm, respectively) or a combination of vanillate and GGE (4 and 1.5 mm, respectively). For *S. xenophagum* cultures, vanillate was replaced by glucose, because we found this strain to be unable to metabolize vanillate. *N. aromaticivorans* was also grown in SMB containing 165 μm GGE. Starter cultures were grown in SMB with 4 mm vanillate or glucose, and cells were pelleted and washed with PBS (10 mm Na_2_HPO_4_, 1.8 mm KH_2_PO_4_, 137 mm NaCl, 2.7 mm KCl, pH 7.4). Pellets were resuspended into culture medium and used to inoculate experimental cultures to initial cell densities of <5 KU.

Cultures were grown aerobically at 30 °C, in 125-ml conical flasks containing 20–40 ml of medium and shaken at ∼200 rpm. Aliquots (400–600 μl) were removed at specified time points and filtered through 0.22-μm syringe tip filters (*e.g.* Whatman Puradisc filters, GE Healthcare) before HPLC analysis of extracellular aromatics. Every culture was grown at least three times; data shown are from representative cultures.

### Enzyme purifications

Saro_2595 from *N. aromaticivorans*, *yghU* and *yfcG* from *E. coli* DH5α, and SLG_04120 (SLG_RS02030 in the recently reannotated genome in NCBI) from *Sphingobium* sp. SYK-6 (codon-optimized for expression in *E. coli*) were individually cloned into plasmid pVP302K (34) so that transcripts from the plasmids would be translated into proteins containing a His_8_ tag connected to the N terminus via a tobacco etch virus (TEV) protease recognition site (construction of plasmids described in the supporting information). The plasmid containing Saro_2595 was modified via PCR to generate plasmids for expressing variants of NaGST_Nu_ containing the single-residue mutations T51A, Y166F, and Y224F. Recombinant proteins were expressed in *E. coli* B834 (35, 36) containing plasmid pRARE2 (Novagen, Madison, WI) grown for ∼25 h at 25 °C in ZYM-5052 autoinduction medium (37) containing kanamycin and chloramphenicol. Recombinant proteins were purified as described previously (15) (see supporting information for modifications to the procedure). After removal of His_8_ tags using TEV protease, recombinant proteins retained a Ser-Ala-Ile-Ala-Gly- peptide on their N termini, derived from the linker between the protein and the TEV protease recognition site. Recombinant LigE and LigF1 from *N. aromaticivorans* were purified as described previously (34).

### Kinetics of converting GS-HPV into HPV

The reaction buffer (RB) consisted of 25 mm Tris-HCl (pH 8.5) and 25 mm NaCl. The β(*R*)- and β(*S*)-stereoisomers of GS-HPV were separately generated by incubating racemic β(*S*)- and β(*R*)-MPHPV (0.46 mm) in RB with 5 mm GSH and either 38 μg/ml LigF1 or 36 μg/ml LigE for several hours (Fig. S4). This sample, containing a single GS-HPV stereoisomer, guaiacol, and the unreacted MPHPV stereoisomer (as well as LigE or F1), was diluted with RB to achieve the desired concentration of GS-HPV for the time course reaction (0.005, 0.01, 0.02, 0.1, or 0.2 mm). An additional 5 mm GSH (dissolved in RB) was added before initiation of each time course. At time 0, 100 μl of the indicated enzyme (resuspended in RB) was combined with 1800 μl of the diluted GS-HPV reaction mixture to achieve final concentrations of 8 nm NaGST_Nu_, 20 nm NaGST_Nu_ (T51A), 100 nm NaGST_Nu_ (Y166F), 10 nm NaGST_Nu_ (Y224F), 195 nm EcYghU, 195 nm EcYfcG, or 47 or 18 nm SYK6GST_Nu_ (for the β(*R*)- and β(*S*)-GS-HPV reactions, respectively). Reactions were performed at 25 °C. At specified time points, 300 μl of the reaction was removed and combined with 100 μl of 1 m HCl (Acros Organics, Geel, Belgium) to stop the reaction before HPLC analysis to quantify HPV formed.

### N. aromaticivorans cell extract assays

*N. aromaticivorans* cells were grown in 500-ml conical flasks containing 267 ml of SMB medium with 4 mm vanillate and 1 mm GGE. When cell densities reached ∼1 × 10^9^ cells/ml, cells were lysed by the sonication procedure used to generate *E. coli* lysates for protein purification (see supporting information). Samples were centrifuged at 7000 × *g* for 15 min, and the supernatants were used as crude cell extracts.

Assays containing 50 mm Tris-HCl (pH 8.0), 50 mm NaCl, 10 mm GSH, 0.407 mm racemic β(*R*)- and β(*S*)-MPHPV and cellular extract from 12444Δ1879 or 12444Δ2595 (final concentrations of 269 and 186 μg of protein/ml, respectively) were performed at 30 °C. At defined time points, 300-μl aliquots were combined with 100 μl of 1 m HCl to stop the reaction before HPLC analysis. At the indicated time, recombinant NaGST_Nu_ (30 μg of protein/ml) was added to the 12444Δ2595 cell extract reaction, along with an additional 10 mm GSH.

### HPLC analysis

After extracellular aromatics were identified using LC-MS with a PFP column (see supporting information), routine analysis and quantification of aromatics were performed using an Ultra AQ C18 5-μm column (Restek) attached to a System Gold HPLC system (Beckman Coulter) with running buffers described in Fig. S11*A*. The eluent was analyzed for light absorbance between 191 and 600 nm, and absorbances at 280 nm were used for quantification of aromatic metabolites by comparing peak areas with those of standards (retention times of measured metabolites are shown in Figs. S4 and S11*B*).

### Production of cDNA libraries from N. aromaticivorans cultures and real-time quantitative PCR

*N. aromaticivorans* cultures were grown in 120 ml of SMB containing either 4 mm vanillate or 4 mm vanillate and 1 mm GGE. Cells were harvested when the vanillate concentration of a culture's medium was ∼20% of its initial value (cell densities of ∼8 × 10^8^ cells/ml); at this point, ∼65% of the GGE initially present in the GGE-fed culture was converted into downstream intermediates. Because both cultures were actively metabolizing vanillate, and only one culture was actively metabolizing GGE, the differences in transcript levels between the cultures should largely be related to GGE metabolism.

40 ml of harvested culture was combined with 5.71 ml of ice-cold stop solution (95% ethanol, 5% acid phenol/chloroform (5:1 solution, pH 4.5)). These mixtures were centrifuged at 4 °C for 12 min at 6000 × *g*. Cell pellets were resuspended into 2 ml of lysis solution (2% SDS, 16 mm EDTA in RNase-free water) and then incubated at 65 °C for 5 min. RNA purification and cDNA synthesis were performed as described previously (38), using SuperScript III reverse transcriptase (Thermo Fisher Scientific) to construct the cDNA library. Genes selected for transcript analysis were the top BLASTp hits in the *N. aromaticivorans* genome of gene products from *Sphingobium* sp. SYK-6 or *Novosphingobium* sp. MBES04 previously shown to catalyze reactions of the β-etherase pathway (Table 1).

Real-time qPCR was performed on a 7500 real-time PCR system (Applied Biosystems, Forest City, CA) using SYBR Green JumpStart Taq ReadyMix (Sigma-Aldrich). Primers used to detect transcripts are contained in Table S5. Transcript levels were normalized to those of Saro_0141 (*rpoZ*, coding for the RNA polymerase Omega subunit).

### Determination of COD

Initial COD values for cultures were obtained either from uninoculated medium or from inoculated medium that was immediately passed through a 0.22-μm filter. Final COD samples were collected when cultures reached their maximum cell densities, both from unfiltered culture (cells and medium) and filtered culture (medium). The difference in COD between the unfiltered and filtered final samples is defined as the COD of cellular biomass. Samples were diluted as needed and combined with High Range COD Digestion Solution (Hach, Loveland, CO). The mixtures were heated to 150 °C for 120 min to oxidize the materials before absorbances were measured at 600 nm. Standards with known COD values were analyzed in parallel.

### Chemicals

Vanillate, guaiacol, GSH, and 2,3-dichloro-5,6-dicyano-*p*-benzoquinone (DDQ) were purchased from Sigma-Aldrich. *erythro*-GGE was purchased from TCI America (Portland, OR).

A racemic mixture of MPHPV was synthesized by dissolving *erythro*-GGE into ethyl acetate (Fisher) and then slowly adding 1.25 molar eq of DDQ and stirring for 30 min. The reaction was washed three times with saturated NaHCO_3_ to remove DDQH_2_ formed during the reaction. The MPHPV was purified via flash chromatography using hexane/ethyl acetate (0.33:0.67, v/v), as described previously (15), and then crystallized from the eluent via solvent evaporation.

HPV was synthesized as described previously for synthesis of β-deoxy-α-veratrylglycerone, except using 4-*O*-benzyl-acetovanillone as starting material, rather than acetoveratrone (15). Synthesis of HPV required an additional debenzylation step that was unnecessary in the synthesis of β-deoxy-α-veratrylglycerone.

### Structure determination

NaGST_Nu_ was screened for crystal formation against several commercial screens at 277 and 293 K using a TTP Labtech Mosquito® crystallization robot. The best-diffracting ammonium acetate–precipitated crystal was obtained at 293 K, by mixing 0.2 μl of protein solution (277 μm protein preincubated for 50 min with 10 mm GSH (neutralized with NaOH)) with 0.2 μl of reservoir solution (4 m ammonium acetate buffered with 100 mm sodium acetate, pH 4.6). This crystal was mounted directly from the growth solution by drawing it through a layer of fomblin oil, thinning the surrounding liquid with a paper wick, and plunging into liquid nitrogen. The best-diffracting ammonium sulfate–precipitated crystal was obtained at 293 K using 0.13 μl of protein solution and 85 nl of reservoir solution (1.35 m ammonium sulfate, 0.1 m lithium sulfate, and 0.1 m Bistris propane, pH 7.5). This crystal was cryopreserved by adding 0.5 μl of a solution composed of 2 parts reservoir solution and 1 part undiluted glycerol to the droplet containing the crystal and equilibrating for 11 min before looping and plunging into liquid nitrogen.

Diffraction data were obtained at the GM/CA beamline at Argonne National Laboratory with an Eiger 16M detector (39). Data were collected on the ammonium acetate (PDB code 5UUN) and ammonium sulfate (PDB code 5UUO) crystal forms using 1.033 Å (for 1.45 Å resolution) or 0.7749 Å (for 1.25 Å resolution) X-rays, respectively. Diffraction data were reduced using XDS (40). Both crystals belonged to space group P2_1_2_1_2_1_ with a predicted solvent content of 60%. The structure was solved by molecular replacement with Phaser (41) in the Phenix suite (42), using a search model based on PDB entry 3C8E:A (EcYghU (18)) modified with phenix.sculptor (43), based on primary sequence alignment. Phenix.refine (44) and COOT (45) were alternatively used to refine the structure and fit the model to electron density maps. Structure solution revealed two copies of the protein per asymmetric unit, with strong electron density present for the paired active-site glutathione molecules. The composite simulated annealing omit map for the active-site glutathiones was calculated in Phenix using default parameters and contoured at 2σ. The active sites were modeled containing either two GSH molecules or a single GSSG molecule, and difference density maps were calculated by setting the occupancy of either conformation to one or zero and then running one round of standard refinement, contoured at 4σ. The omit map data were best fit to a model in which each active site contained electron density from both a pair of GSHs (locked to be of equal occupancy, Q(GSHA) = Q(GSHB)) and a single GSSG (occupancy 1-Q(GSHA,B)), which was interpreted as a heterogeneous population of protein molecules containing either two GSHs or one GSSG throughout the crystal (Fig. S6).

### Molecular modeling of substrate binding

For modeling β(*R*)- and β(*S*)-GS-HPV and their syringyl phenylpropanoid analogues into the active sites of NaGST_Nu_ and EcYghU, the PyMOL Builder function (46) was used to create molecules of GS-HPV or GS-syringyl by adding atoms onto the GSH2 molecule bound in each active site. Atoms were added so as to visually minimize steric clash with the proteins. The potential energies of the protein-GS-phenylpropanoid complexes were minimized using the Minimize Structure function of UCSF Chimera (23). For the energy minimization, all of the atoms of the protein-GS-phenylpropanoid complex were held rigid except for those of the phenylpropanoid moiety and the Cys side chain of GSH2. 100 steepest descent steps were run, followed by 20 conjugate gradient steps, and all step sizes were 0.05 Å.

## Author contributions

W. S. K., D. R. N., and T. J. D. conceptualization; W. S. K., C. A. B., C. N. O., D. R. W., A. U., and L. M. Y. data curation; W. S. K., C. A. B., C. N. O., D. R. W., and B. G. F. formal analysis; W. S. K., C. N. O., D. R. W., and L. M. Y. investigation; W. S. K., C. N. O., A. U., D. L. G., and R. W. S. methodology; W. S. K., B. G. F., and T. J. D. writing-original draft; W. S. K., B. G. F., D. R. N., and T. J. D. writing-review and editing; B. G. F. and T. J. D. resources; B. G. F., D. R. N., and T. J. D. supervision; B. G. F., D. R. N., J. J. C., and T. J. D. funding acquisition; B. G. F. visualization; T. J. D. project administration; C. A. B. and R. W. S. crystallized NaGSTNu and solved its structure; A. U. and J. J. C. identified metabolites via LC-MS; D. L. G. purified LigE and LigF and synthesized MPHPV and HPV.

## Supplementary Material

Supporting Information

## Footnotes

3 The residue numbers used here for NaGST_Nu_ are for the native protein; residue numbers in the PDB entries differ by +5 because the protein used for crystallization contained an N-terminal extension left after proteolytic removal of the His_8_ tag.

## Footnotes

2 The abbreviations used are:

GGE: guaiacylglycerol-β-guaiacyl ether
GST: glutathione *S*-transferase
MPHPV: β-(2-methoxyphenoxy)-γ-hydroxypropiovanillone
GS-HPV: β-glutathionyl-γ-hydroxypropiovanillone
HPV: hydroxypropiovanillone
SG: conjugated glutathione moiety
COD: chemical oxygen demand
TEV: tobacco etch virus
PDB: Protein Data Bank
r.m.s.: root mean square
SMB: standard mineral base
RB: reaction buffer
DDQ: 2,3-dichloro-5,6-dicyano-*p*-benzoquinone
KU: Klett unit
Bistris propane: 1,3-bis[tris(hydroxymethyl)methylamino]propane.
