# Supplementary material for: Novosphingobium aromaticivorans uses a Nu-class glutathione S-transferase as a glutathione lyase in breaking the β-aryl ether bond of lignin
Source: J Biol Chem. 2018 Feb 15;293(14):4955–68. doi: 10.1074/jbc.RA117.001268 (PMC5892560; doi:10.1074/jbc.RA117.001268)
Supplement: Supporting Information [file supp_293_14_4955__index.html]

Novosphingobium aromaticivorans uses a Nu-class glutathione S-transferase as a glutathione lyase in breaking the β-aryl ether bond of lignin — Deglutathionylation of a GS-phenylpropanoid by Nu-class GSTs — Supporting Information 

# *Novosphingobium aromaticivorans* uses a Nu-class glutathione *S*-transferase as a glutathione lyase in breaking the β-aryl ether bond of lignin

## Supporting Information

- Supporting Information - Supporting Information (Figures, Methods, etc.)
