# Supplementary material for: Novosphingobium aromaticivorans uses a Nu-class glutathione S-transferase as a glutathione lyase in breaking the β-aryl ether bond of lignin
Source: J Biol Chem. 2018 Feb 15;293(14):4955–68. doi: 10.1074/jbc.RA117.001268 (PMC5892560; doi:10.1074/jbc.RA117.001268)
Supplement: Supporting Information [file supp_RA117.001268_134384_1_supp_67035_p3hjrc.pdf]

SUPPORTING INFORMATION

Novosphingobium aromaticivorans uses a Nu-class glutathione-S-transferase as a glutathione lyase in breaking the β-aryl ether bond of lignin

Wayne S. Kontur[1,2], Craig A. Bingman[2,3], Charles N. Olmsted[1,2], Douglas R. Wassarman[1,2], Arne Ulbrich[4,5], Daniel L. Gall[1,2], Robert W. Smith[2,3], Larissa M. Yusko[4], Brian G. Fox[1,2,3], Daniel R. Noguera[1,2,6], Joshua J. Coon[1,2,4,5,7] and Timothy J. Donohue[1,2,8,*]

Affiliations: [1]Wisconsin Energy Institute, University of Wisconsin-Madison, Madison, WI; [2]DOE Great Lakes Bioenergy Research Center, University of Wisconsin-Madison, Madison, WI; [3]Department of Biochemistry, University of Wisconsin-Madison, Madison, WI; [4]Department of Chemistry, University of Wisconsin-Madison, Madison, WI; [5]Genome Center of Wisconsin, University of Wisconsin-Madison, Madison, WI; [6]Department of Civil & Environmental Engineering, University of Wisconsin-Madison, Madison, WI; [7]Department of Biomolecular Chemistry, University of Wisconsin-Madison, Madison WI; [8]Department of Bacteriology, University of Wisconsin-Madison, Madison, WI

*To whom correspondence should be addressed: Timothy J. Donohue, Department of Bacteriology, University of Wisconsin-Madison, Madison, WI; E-mail: tdonohue@bact.wisc.edu; Phone: (608) 262-4663

Table of Contents

Figure S1……………………………………………………………………………page S-3
Table S1……………………………………………………………………………page S-4
Figure S2……………………………………………………………………………page S-5
Figure S3……………………………………………………………………………page S-6
Figure S4……………………………………………………………………………page S-7
Figure S5……………………………………………………………………………page S-8
Figure S6……………………………………………………………………………page S-9
Figure S7……………………………………………………………………………page S-10
Figure S8……………………………………………………………………………page S-11
Figure S9……………………………………………………………………………page S-12
Table S2……………………………………………………………………………page S-13
Recipe for media components……………………………………………………page S-14
Table S3……………………………………………………………………………page S-15
Construction of *N. aromaticivorans* mutants…………………………………page S-17
Figure S10…………………………………………………………………………page S-19
Table S4……………………………………………………………………………page S-20
Expression and purification of recombinant proteins…………………………page S-21
Identification and quantification of extracellular metabolites………………page S-23
Figure S11…………………………………………………………………………page S-24
Table S5……………………………………………………………………………page S-25
References…………………………………………………………………………page S-26

Figure S1: Growth and extracellular metabolite levels in a representative culture of *Novosphingobium aromaticivorans* 12444Δ1879 in SMB containing 165 μM GGE.

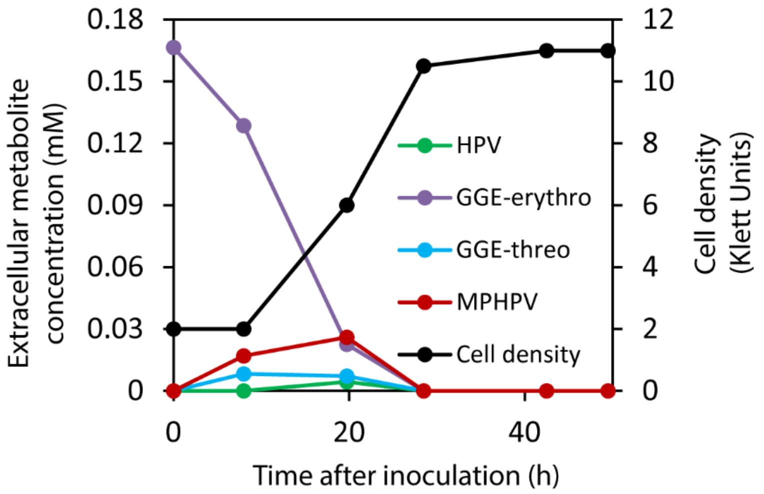

Table S1: Chemical oxygen demand (COD) analysis of bacterial cultures[a]

Strain	Carbon sources	Initial COD[b]	Final COD (biomass)[c]	Final COD (soluble)[d]	%COD incorporated into biomass[e]	%COD lost from the culture[f]
N. aromaticivorans 12444Δ1879	3 mM GGE (Fig. 2A,B)	2100 ± 100	480 ± 70	500 ± 100	22%	53%
	4 mM vanillate (Fig. 2G)	1300 ± 100	530 ± 40	280 ±80	41%	37%
	4 mM vanillate, 1.5 mM GGE (Fig. 2G,H)	2170 ± 80	720 ± 80	340 ± 60	33%	51%
Novosphingobium sp. PP1Y	4 mM vanillate (Fig. S2E)	1200 ± 100	420 ± 20	240 ± 30	36%	44%
	4 mM vanillate, 1.5 mM GGE (Fig. S2E,F)	2070 ± 90	420 ± 20	1080 ± 20	20%	28%
S. xenophagum NBRC 107872	4 mM glucose (Fig. S2G)	1040 ± 30	490 ± 30	340 ± 20	47%	20%
	4 mM glucose, 1.5 mM GGE (Fig. S2G,H)	1970 ± 50	460 ± 30	1270 ± 30	23%	12%
N. aromaticivorans 12444Δ2595	3 mM GGE (Fig. 2C,D)	2090 ± 40	30 ± 100	1900 ± 300	1%	6%
	4 mM vanillate (Fig. 2I)	1300 ± 200	550 ± 90	330 ± 70	41%	34%
	4 mM vanillate, 1.5 mM GGE (Fig. 2I,J)	2200 ± 100	520 ± 80	1200 ± 100	23%	24%
N. aromaticivorans 12444EcyghU	3 mM GGE (Fig. 2E,F)	2200 ± 90	500 ± 100	500 ± 100	23%	52%
	4 mM vanillate (Fig. 2K)	1400 ± 300	570 ± 50	430 ± 50	42%	26%
	4 mM vanillate, 1.5 mM GGE (Fig. 2K,L)	2400 ± 100	600 ± 100	450 ± 80	26%	55%

[a]Units of COD are mg/L.
[b]Initial COD is that of the medium before inoculation or inoculated medium that has immediately been filtered.
[c]Final COD (biomass) is the difference between the unfiltered and filtered final samples.
[d]Final COD (soluble) is the COD remaining in the medium after filtering the final sample.
[e]%COD incorporated into biomass is the ratio of Final COD (biomass) to Initial COD.
[f]%COD lost = 1 – (Final COD (biomass) + Final COD (soluble))/Initial COD. It is assumed that the lost COD represents the electrons in the system that were combined with oxygen during cell growth.

Figure S2: Growth and extracellular metabolite concentrations from representative cultures of *Novosphingobium* sp. PP1Y (A,B,E,F) and *Sphingobium xenophagum* (C,D,G,H) grown in SMB containing 3 mM GGE (A,B,C,D); or 1.5 mM GGE with 4 mM vanillate (E,F) or glucose (G,H). The y-axis segments of (D,F,H) use different concentration scales. For comparison, cell densities for cultures grown in SMB containing only 4 mM vanillate or glucose are included in panels E and G.

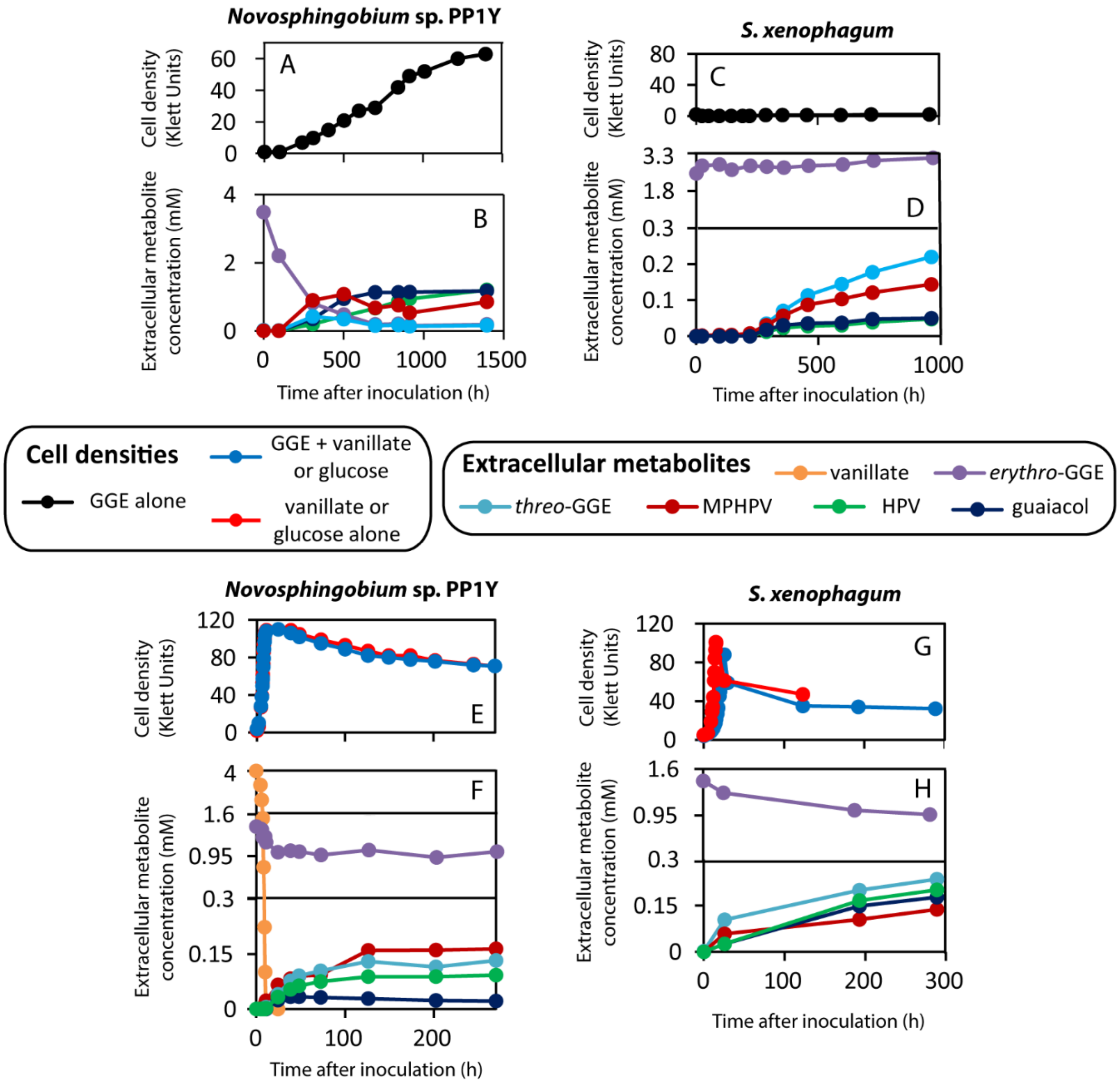

Figure S3: Amino acid sequence alignment of Nu-class GSTs NaGST$_{\mathrm{Nu}}$ (*N. aromaticivorans*), SYK6GST$_{\mathrm{Nu}}$ (*Sphingobium* sp. SYK-6), EcYghU (*E. coli*), MBENS4_4395 (*Novosphingobium* sp. MBES04), SsYghU (*S. sanguinis*), GST3 (*Novosphingobium* sp. MBES04), and EcYfcG (*E. coli*). Enzymes whose structure are solved are underlined. Residues identified from structures to interact with either GSH or GSSG are highlighted in yellow. Residues in NaGST$_{\mathrm{Nu}}$ proposed in the main text to interact with GS-HPV during the glutathione lyase reaction are highlighted in green. Alignment was made using Clustal Omega (1.2.4) (http://www.ebi.ac.uk/Tools/msa/clustalo/).

```
NaGSTNu          ---------MSSEYVPPKVWKWDKANGGAFASVNRPVAGPTSERELPVGKHPFQVYSLGT         51
SYK6GSTNu        MADSDPSMNQPTGYVPPKVWTWDKENGGQFSNINAPTAGARQDVTLPVGEHPIQLYSLGT         60
EcYghU           --------MTDNTYQPAKVWTWDKSAGGAFANINRPVSGPTHEKTLPVGKHPLQLYSLGT         52
MBENS4_4395      --------MADTTYTPPKVWTHDAENGGTWASINRPTAGAREEKELPRGEHPFQLYSLGT         52
SsYghU           -----------MTYQLPKVWSAADSDQGKFSGINQPTAGVRFEQKLPVGKEPFQLYSLGT         49
GST3             ---------------------------------------------------MLELWTSET         9
EcYfcG           ---------------------------------------------------MIDLYFAPT         9
                                                                    ::::   *

NaGSTNu          PNGQKATIMLEELLQLGFSEAEYDAWLIKIFEGDQFTSGFVDINPNSKIPAMVDRS---G         108
SYK6GSTNu        PNGQKVTIMLEELLAAGF-DAEYDAWLIKIYTGEQFGSDFVAINPNSKIPAMMDHG---L         116
EcYghU           PNGQKVTIMLEELLALGVTGAEYDAWLIRIGDGDQFSSGFVEVNPNSKIPALRDHT---H         109
MBENS4_4395      PNGQKVTILFEELLAAGVSEAEYDAWLVNIGEGTQFTSGFVEINPNSKIPALLDCS---G         109
SsYghU           PNGVKVTIMLEELLAAGVTEATYDLYKISIMDGDQFGSDFVKINPNSKIPALLDQS---G         106
GST3             PNGWKTTIMLEEL------DANYTLRPISLTNREQKEDWYLARNPNGRIPTLIDHEVDAG         63
EcYfcG           PNGHKITLFLEEA------ELDYRLIKVDLGKGGQFRPEFLRISPNNKIPAIVDHSPADG         63
                 *** * *:::**          *    : :    *    ::  .**.:**:: *

NaGSTNu          PEPFRVFESGAILMHLAEKFGVFLPTSGPARAECLSWLFWQVGSAPFIGGGFGHFYNYAP         168
SYK6GSTNu        DPPLRLFESGSMLVYLAEKFGAFLPTEIRKRTETFNWLMWQMGSAPFVGGGFGHFYAYAP         176
EcYghU           NPPIRVFESGSILLYLAEKFGYFLPQDLAKRTETMNWLFWLQGAAPFLGGGFGHFYHYAP         169
MBENS4_4395      ETPVRVFESGAILFHLAEKFGMFLPKDGAARTECMSWLMWQMSTAPIIGGGFGHFYAYAP         169
SsYghU           HKPIPVFESANILLYLAEKFGKLIPSDLAGRTEVLNWLFWQTGAAPFLGGGFGHFFNYAP         166
GST3             NGGFAVFESGAILIYLAEKFGRFLPADTMGRSRAIQWVMWQMSGLGPMMGQATVFNRYFE         123
EcYfcG           GEPLSLFESGAILLYLAEKTGLFLSHETRERAATLQWLFWQVGGLGPMLGQNHHFNHAAP         123
                    . :***. :*.:**** * ::  .   *:  :.*::*  .    : *    *

NaGSTNu          IKIEYAIDRYAMETKRLFDVANRRLAESRYLAGDEYTIADLATYTWFGNIYRGEAYGEAA         228
SYK6GSTNu        FKIEYAIDRYAMETKRQLDVLDKNLADREFMIGDEITIADFAIFPWYGSIMRGGY--NAQ         234
EcYghU           VKIEYAINRFTMEAKRLLDVLDKQLAQHKFVAGDEYTIADMAIWPWFGNVVLGGVYD-AA         228
MBENS4_4395      EKFEYPINRFAMETKRIMDVADKRLAETRYLAGDDYTIADIAAYGWLGGFVRGESYTDAA         229
SsYghU           EKLEYPINRFTMEAKRQLDLLDKELAKKAYIAGEDYSIADIAIWSWYGQLVQDKLYPGAA         226
GST3             PRLPEVIDRYTRESRRLFEVMDTHLADNEFLAG-DYSIADIACFPWVRGHDW--------         174
EcYfcG           QTIPYAIERYQVETQRLYHVLNKRLENSPWLGGENYSIADIACWPWVNAWTR--------         175
                   :   *:*:  *::*  .: : .* .  :: * : :***:* : *

NaGSTNu          TFLSMHEYEHVGRWVGEIDARPGVLRGRLVNSSK-----GLAERHDASDFDALPPESLQA         283
SYK6GSTNu        EFLSTHEYRNVDRWVTQLSERTGVKRGLLVNSAGRP-GGGIAERHSAADLDASIKAAEQE         293
EcYghU           EFLDAGSYKHVQRWAKEVGERPAVKRGRIVNRTNGPLNEQLHERHDASDFETNTEDKRQG         288
MBENS4_4395      TFLSVHEYTNLMRWGEELGERTGVKRGLVVNRDA---PGKIKERHSAADIDAVLGA----         282
SsYghU           EFLDAASYKHLSAWAEKIAARPAVQRGLAAEYQEIK------------------------         262
GST3             ACIDMEGLPHLQRWFETIGERPAVQRGLLLPEPPKAD--EMAEKTTRQ------GKN---         223
EcYfcG           QRIDLAMYPAVKNWHERIRSRPATGQALLKAQLGDER----SDS----------------         215
                   :.      :  *   :  * .. :.

NaGSTNu          IVKGF-      288
SYK6GSTNu        AAKTEA      299
EcYghU           ------      288
MBENS4_4395      ------      282
SsYghU           ------      262
GST3             ILA---      226
EcYfcG           ------      215
```

Figure S4: Representative HPLC data (absorbances at 280 nm) showing generation of β(*R*)- and β(*S*)-GS-HPV from racemic MPHPV and conversion of β(*R*)- and β(*S*)-GS-HPV into HPV by $NaGST_{Nu}$. (A) Racemic MPHPV. (B) Racemic MPHPV combined with LigF and GSH for several hours to generate β(*R*)-GS-HPV and guaiacol. (C) $NaGST_{Nu}$ and additional GSH are then added to convert the β(*R*)-GS-HPV into HPV. (D) Racemic MPHPV combined with LigE and GSH for several hours to generate β(*S*)-GS-HPV and guaiacol. (E) $NaGST_{Nu}$ and additional GSH are then added to convert the β(*S*)-GS-HPV into HPV. Note that different stereoisomers of MPHPV and GS-HPV are not separated in our HPLC system.

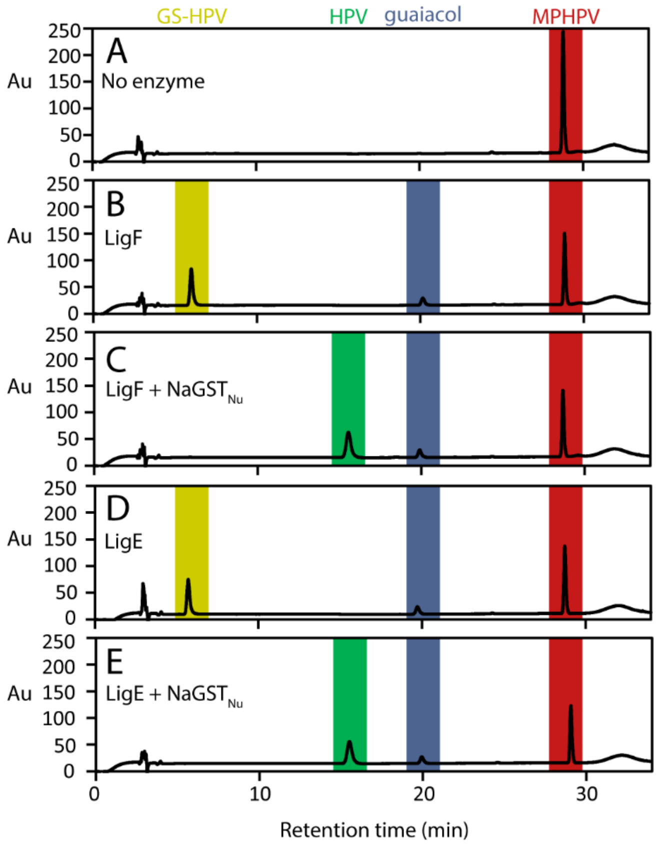

Figure S5: Kinetics of the conversion of β(*R*)- and β(*S*)-GS-HPV into HPV by bacterial Nu-class GSTs. Reactions used 8 nM $NaGST_{Nu}$ (A), 20 nM $NaGST_{Nu}$ (T51A) (B), 100 nM $NaGST_{Nu}$ (Y166F) (C), 10 nM $NaGST_{Nu}$ (Y224F) (D), 47 nM or 18 nM $SYK6GST_{Nu}$ (for the β(*R*)- and β(*S*)-GS-HPV reactions, respectively) (E), 195 nM EcYghU (F), or 195 nM EcYfcG (G). Lines are non-linear least squares best fits to the experimental data using the Michaelis-Menten equation.

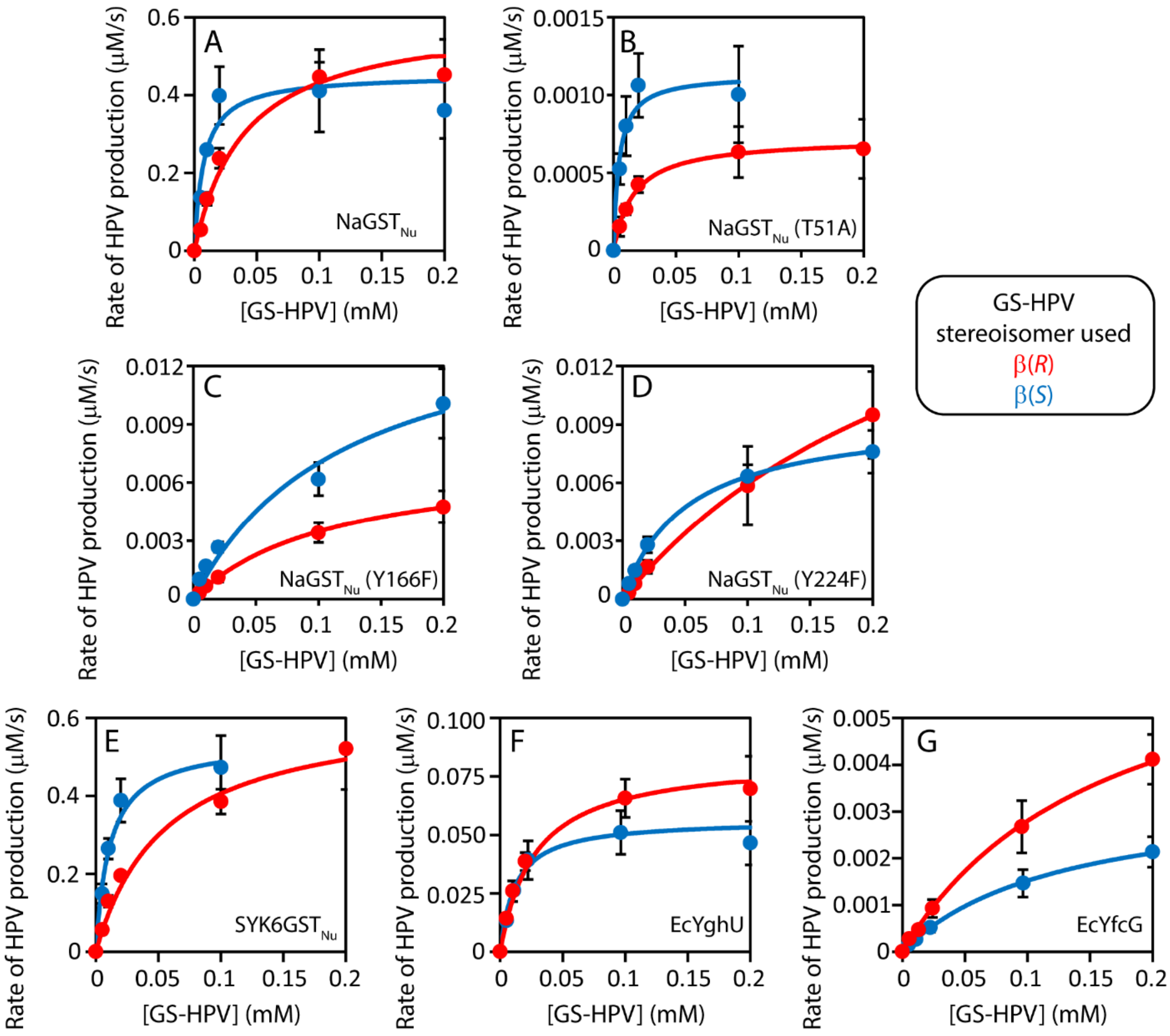

Figure S6: Electron density omit maps for the active site of a $NaGST_{Nu}$ subunit. (A) Composite simulated annealing omit map. (B) Difference density map for a model in which the active site contains two GSH molecules. Negative density is shown in red; positive density is shown in green. (C) Difference density map for a model in which the active site contains a single GSSG molecule. Negative density is shown in magenta; positive density is shown in green. Difference density maps show differences in electron density for the active site models relative to the composite electron density map. The best fit to the composite electron density data was a model in which the active sites contained a mixed population of ~60% GSH-GSH and ~40% GSSG. To explain this, we propose that, prior to diffraction, each enzyme subunit was bound to a GSSG molecule. As the crystal, which was substantially larger than the diffraction beam, was rotated throughout the diffraction process, the bound GSSG molecules near the center of rotation were likely reduced to GSHs by photoelectrons generated by the X-rays. However, new crystal material, still containing GSSG molecules, was continuously rotated into diffracting position, leading to diffraction data for both glutathione configurations.

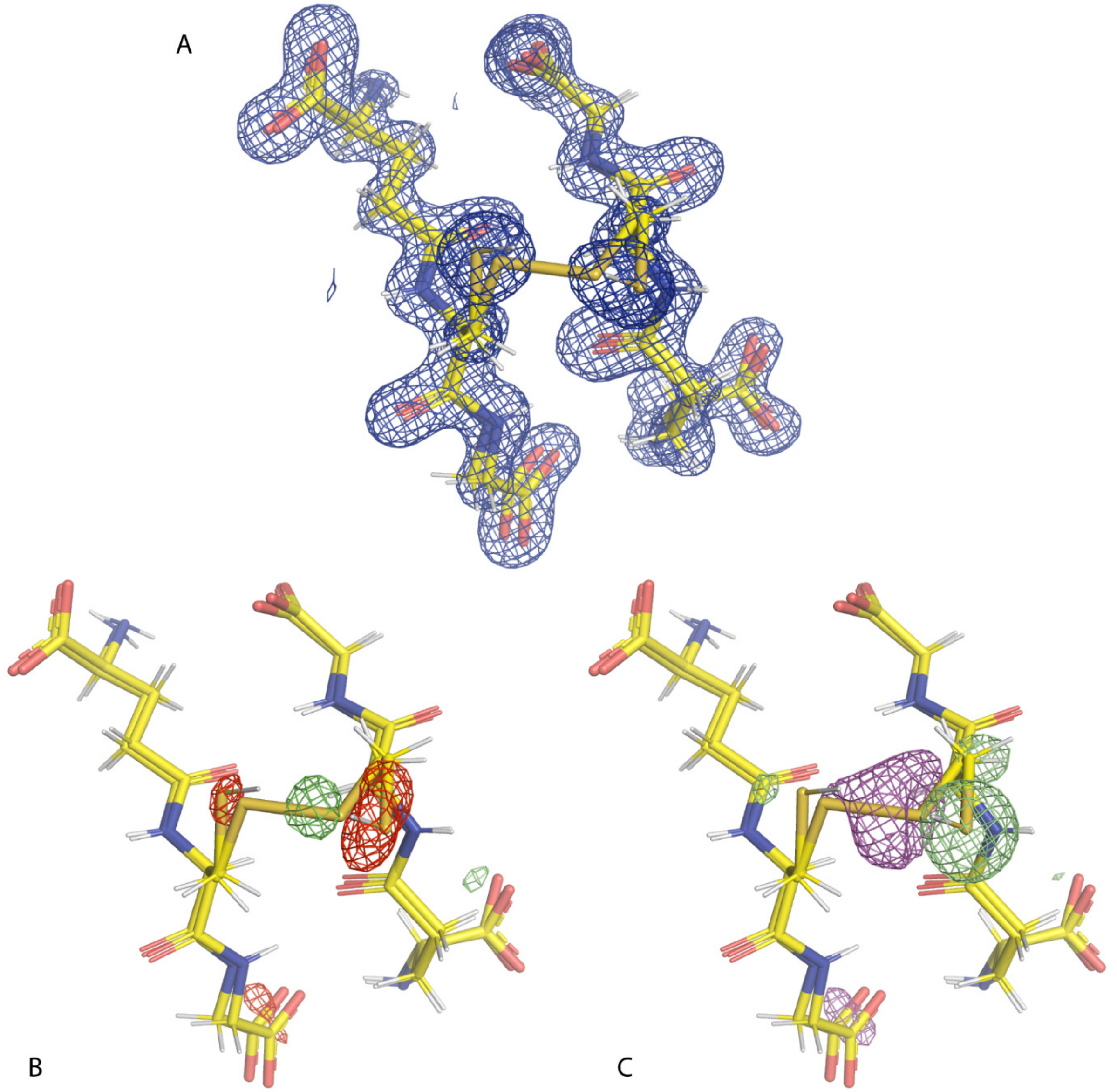

Figure S7: Modeling of substrates into the active sites of $NaGST_{Nu}$ and EcYghU. Panels (A) and (B) show modeling of β(*R*)- and β(*S*)-GS-HPV into $NaGST_{Nu}$. Panels (C) and (D) show modeling of β(*R*)- and β(*S*)-GS-conjugated syringyl phenylpropanoids into $NaGST_{Nu}$. Panels (E) and (F) show modeling of β(*R*)- and β(*S*)-GS-HPV into EcYghU. Coloring for $NaGST_{Nu}$ is the same as in Fig. 4 in the main text (with residues for EcYghU in parentheses): E4 (T5) to P38 (P39) in gray; V39 (V40) to G129 (G130) in green; V130 (Y131) to T134 (Q135) in gray; S135 (D136) to L257 (I257) in maroon; V258 (V258) to F288 (G288) in gold. Residues predicted to be involved in catalysis of the glutathione lyase reaction are Tyr66 and Tyr224 (Tyr167 and Tyr225 in EcYghU). Resides that contribute to differences in active site channel interiors between $NaGST_{Nu}$ and EcYghU are Phe82 and Phe288 in $NaGST_{Nu}$, and Arg260 and Asn262 in EcYghU. Carbon atoms of GSH1 are yellow, and those of the GS-conjugated substrates (GS-HPV or the syringyl analogue) are cyan.

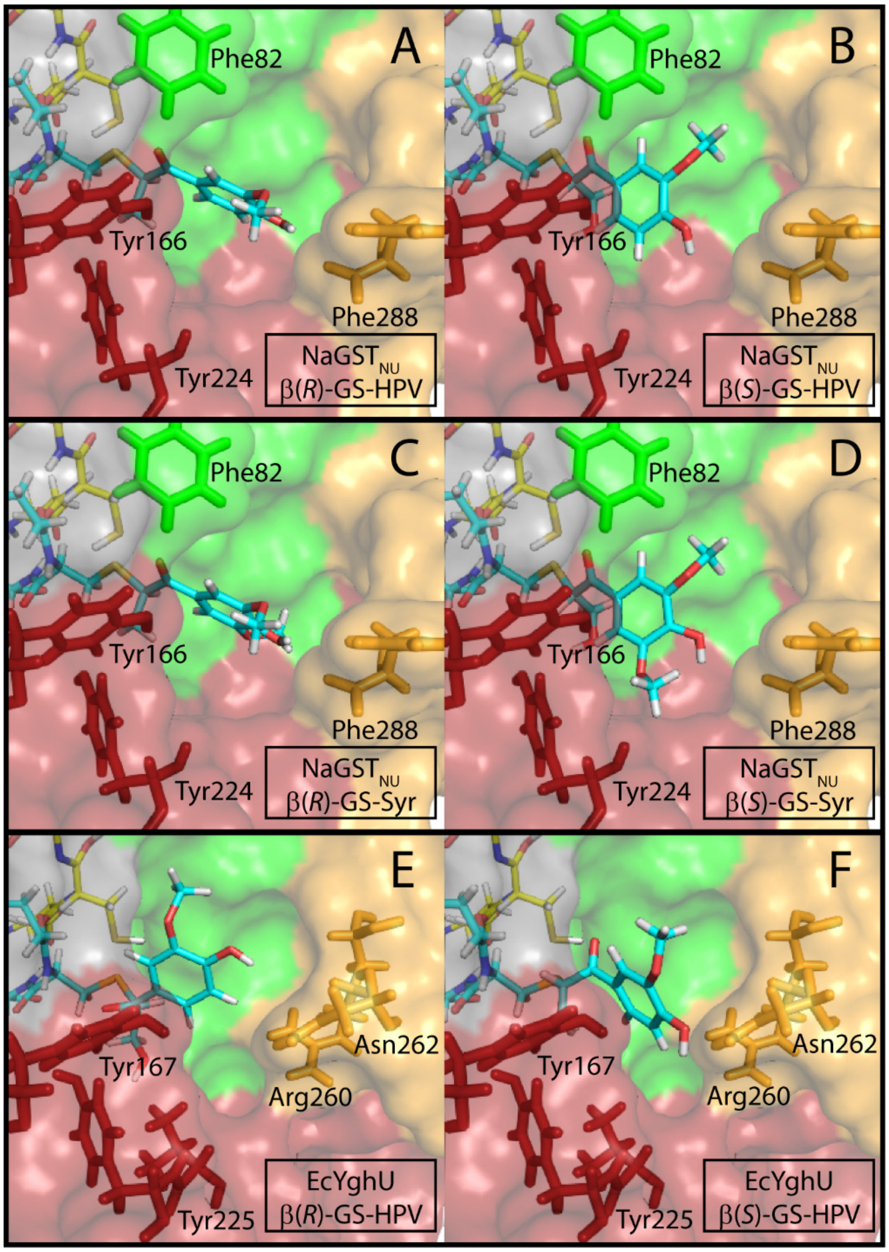

Figure S8: Second-sphere interactions predicted to contribute to the stabilization of the reactive GSH1 thiolate ion by Thr51 and Asn53 in $NaGST_{Nu}$.

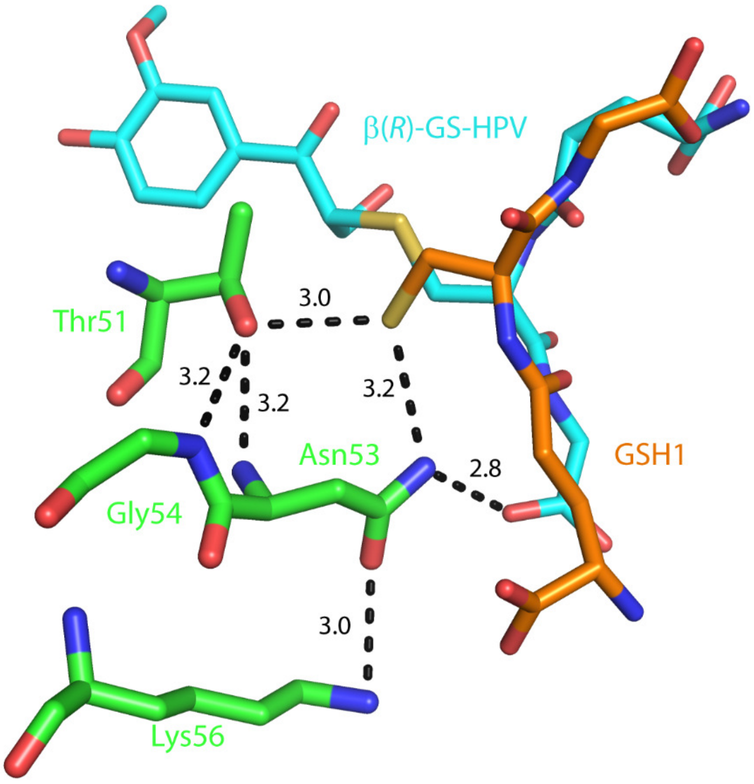

Figure S9: Phylogenetic analysis of Nu-class glutathione-S-transferases. BLASTp searches of the NCBI non-redundant protein database were performed using NaGST$_{Nu}$ and GST3 as queries. The top 5,000 hits from both of these searches were collected; every fifth member from each of these sets was transferred into a new combined set of 2,000 proteins. Sequences for SYK6GST$_{Nu}$ and EcYfcG were added to the combined set, to give a set of 2,002 proteins. Proteins in this set were aligned using MAFFT in MegAlign Pro, which is part of the Lasergene 14.0 suite (DNASTAR, Madison, WI). A phylogenetic tree was calculated via the maximum likelihood method in RAxML v8.2.3 (1), using 100 rapid bootstrap inferences. The tree was visualized using Interactive Tree of Life v3 (http://itol.embl.de). Enzymes experimentally reported here (NaGST$_{Nu}$, SYKGST$_{Nu}$, EcYghU, EcYfcG) or elsewhere (GST3 (2)) to be able to convert GS-HPV into HPV are identified.

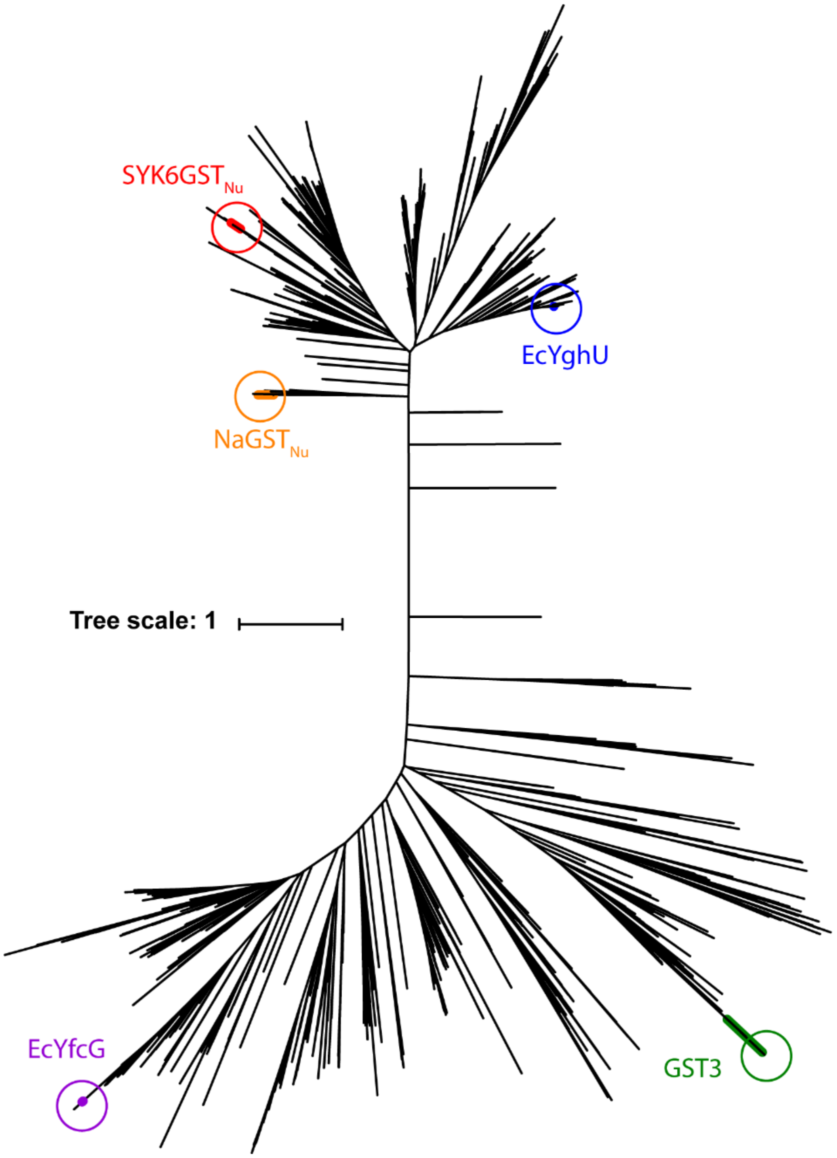

Table S2: Bacterial strains and plasmids used

Strains	Relevant characteristics	References
Novosphingobium aromaticivorans strains		
DSM 12444	Wild-type; also called F199	(3, 4)
12444Δ1879	DSM 12444 ΔSaro_1879	This study
12444Δ2595	12444Δ1879 ΔSaro_2595	This study
12444EcyghU	12444Δ1879 containing *E. coli yghU* at the Saro_2595 locus	This study
Novosphingobium sp. PP1Y	Wild-type	(5)
Sphingobium xenophagum NBRC 107872	Wild-type; also called BN6^T and DSM 6383^T	(6, 7)
Escherichia coli strains		
DH5α	F- Φ80*lac*ZΔM15 Δ(*lac*ZYA-*arg*F) U169 *rec*A1 *end*A1 *hsd*R17 (rK–, mK+) *pho*A *sup*E44 λ– *thi*-1 *gyr*A96 *rel*A1	Bethesda Research Laboratories (8)
S17-1	recA pro hsdR RP4-2-Tc::Mu-Km::Tn7	(9)
Turbo	F' *proA*$^+$*B*$^+$ *lacI*^{q} Δ*lacZM15 / fhuA2* Δ*(lac-proAB) glnV galK16 galE15 R(zgb-210::Tn10)*Tet^S *endA1 thi-1* Δ*(hsdS-mcrB)5*	New England Biolabs
NEB 5-alpha Competent *E. coli*	*fhuA2* Δ(*argF-lacZ*)U169 *phoA glnV44* Φ80 Δ*(lacZ)*M15 *gyrA96 recA1 relA1 endA1 thi-1 hsdR17*	New England Biolabs
E. cloni 10G	F- *mcrA* Δ(*mrr-hsd*RMS-*mcr*BC) *end*A1 *rec*A1 Φ80*dlac*ZΔM15 Δ*lac*X74 *ara*D139 Δ(*ara,leu*)7697*gal*U *gal*K *rps*L *nup*G λ- *ton*A (StrR)	Lucigen
B834	F- *hsdS metE gal ompT*	(10, 11)
**Plasmids**		
pK18*mobsacB*	pMB1ori *sacB kan*^{R} mobT oriT(RP4) *lacZα*	(12)
pK18msB-MCS1	pK18*mobsacB* lacking the multiple cloning site	This study (Supporting Information)
pVP302K	lac promoter lacI, Tev site *rtxA* (*V. cholera*) *kan*^{R}; coding sequence for 8×His-tag	Supplemental Materials of (13)
pRARE2	p15a ori *camR*; tRNA genes for 7 rare codons in *E. coli*	Novagen
pK18msB/ΔSaro1879	pK18*mobsacB* containing genomic regions flanking Saro_1879	This study (Supporting Information)
pK18msB/ΔSaro2595	pK18*mobsacB* containing genomic regions flanking Saro_2595	This study (Supporting Information)
pK18msB/EcyghU-Δ2595	pK18*mobsacB* containing *E. coli yghU* between the Saro_2595 flanking regions	This study (Supporting Information)
pVP302K/Ctag-2595	pVP302K containing Saro_2595 upstream of *rtxA* and His-tag sequence	This study (Supporting Information)
pVP302K/Untagged2595	pVP302K containing Saro_2595	This study (Supporting Information)
pVP302K/Ntag-2595	pVP302K containing Saro_2595 downstream of His-tag coding sequence and Tev protease site	This study (Supporting Information)
pVP302K/Ntag-2595-T51A	Modified pVP302K/Ntag-2595 for expressing $NaGST_{Nu}$ variant T51A	This study (Supporting Information)
pVP302K/Ntag-2595-Y166F	Modified pVP302K/Ntag-2595 for expressing $NaGST_{Nu}$ variant Y166F	This study (Supporting Information)
pVP302K/Ntag-2595-Y224F	Modified pVP302K/Ntag-2595 for expressing $NaGST_{Nu}$ variant Y224F	This study (Supporting Information)
pVP302K/Ntag-EcyghU	pVP302K containing *yghU* downstream of His-tag coding sequence and Tev protease site	This study (Supporting Information)
pVP302K/Ntag-EcyfcG	pVP302K containing *yfcG* downstream of His-tag coding sequence and Tev protease site	This study (Supporting Information)
pVP302K/Ntag-SLG_04120	pVP302K containing SLG_04120 downstream of His-tag coding sequence and Tev protease site	This study (Supporting Information)

Recipes for media components

Hutner's vitamin-free Concentrated Base (from (14))

Per 500 mL:

5 g Nitrilotriacetic acid

14.78 g $MgSO_4 \cdot 7H_2O$

1.67 g $CaCl_2 \cdot 2H_2O$

4.625 mg $(NH_4)_6Mo_7O_{24} \cdot 4H_2O$

49.5 mg $FeSO_4 \cdot 7H_2O$

25 mL Metals "44"

Metals "44"

Per 500 mL:

1.25 g EDTA (free acid) [Add this first; use 10 M NaOH to help dissolve it.]

5.475 g $ZnSO_4 \cdot 7H_2O$

2.5 g $FeSO_4 \cdot 7H_2O$

0.77 g $MnSo_4 \cdot H_2O$

0.196 g $CuSO_4 \cdot 5H_2O$

0.125 g $Co(NO_3)_2 \cdot 6H_2O$

0.0885 g $Na_2B_4O_7 \cdot 10H_2O$

Table S3: Primers used to modify the *N. aromaticivorans* genome and to create recombinant enzyme expression vectors

Name	Sequence	Notes
pK18msB AseI ampl F	5’-CTGTCGTGCCAGCTGCATTAATG-3’	AseI site (underlined) native to template
pK18msB –MCS XbaI R	5’-GAACAtcTAGAAAGCCAGTCCGCAGAAAC-3’	XbaI site (underlined); lowercase bases do not match template
Saro1879 lvnsucr ampl F AseI	5’-CCCGAattaATCGTGACGGTATCAACCTCC-3’	AseI site (underlined); lowercase bases do not match template
Saro1879 lvnsucr ampl R XbaI	5’-GTTTCGGtCtAGATCGAGCTGACCGAAATC-3’	XbaI site (underlined); lowercase bases do not match template
Saro_2595 amp AseI for	5’-GTCGatTAatAGTCCGAGATCGAGGCTGC-3’	AseI site (underlined); lowercase bases do not match template
Saro_2595 amp XbaI rev	5’-CGACtctAGaCAGAGCCTGAACGAAGTC-3’	XbaI site (underlined); lowercase bases do not match template
Saro1879 lvnsucr del REV	5’-CCGACTTTCTTGAAACAGATTTGGCTTAAGAC-3’	
Saro1879 lvnsucr del FOR	5’-GTTCATGCTTAACTTCGATGGCGAGC-3’	
Saro_2595 del rev	5’-CCTGCTCCTTGGGGATATTGTTAGTGTTG-3’	
Saro_2595 del for	5’-GGAATCGTTGCAAGCGATCGTCAAG-3’	
D2595pK18-EcYghU F	5’-GGAGCAGGCGATGACAGACAATACTTATCAGCCCGCGAAAG-3’	underlined region matches sequence in pK18msB/ΔSaro2595
D2595pK18-EcYghU R	5’-CGAGGCGGGTTTACCCCTGACGCTTATCTTCCGTATTCGTC-3’	underlined region matches sequence in pK18msB/ΔSaro2595
EcYghU-D2595pK18 F	5’-TCAGGGGTAAACCCGCCTCGAGACCGGCGAAC-3’	underlined region matches sequence in *yghU*
EcYghU-D2595pK18 R	5’-TGTCTGTCATcgCCTGCTCCTTGGGGATATTGTTAGTG-3’	underlined region matches sequence in *yghU*; lowercase bases are not present in pK18msB/ΔSaro2595, but are present in the *N. aromaticivorans* genome
Saro2595 Ctag PciI F	5’-GCAGGacATGTCCTCAGAGTACGTTCC-3’	PciI site (underlined); lowercase bases do not match template
Saro2595 Ctag BsaI R	5’- GTTatctgcgagaccACGATCGCTTGCAACGATTC-3’	BsaI site (underlined); lowercase bases do not match template
pVP302K Ctag BsaI F	5’-CTGCGGTCTCGCAGATGGTAAAATTCTG-3’	BsaI site (underlined)
pVP302K Ctag NcoI R	5’- GGTGATGTCCCATGGTTAATTTCTCCTCTTTAATG-3’	NcoI site (underlined)
Ctag 2595-pVP add Stop R	5’-tcagaagcccttgACGATCGCTTGCAACGATTC-3’	lowercase bases do not match pCtag-2595/pVP302K; underlined bases are stop codon
pVP302K Ntag HindIII F	5’- CATTAAaAGcTTAAACGAATTCGGACTCGGTACGC -3’	HindIII site (underlined); lowercase bases do not match template
2595-pVP C to Ntag F	5’-caagcgaaaatctgtattttcagagcgcgatcgcagga**ATG**TCCTCAGAGTACGTTCC-3’	lowercase bases do not match template; bold ATG is Saro_2595 start site; underlined region is Tev protease recognition site

pVP302 C to Ntag R	5’-ccaatgcatggtgatggtgatgatggtgatgtcccatGGTTAATTTCTCCTCTTTAATG-3’	lowercase bases do not match template; compliment of coding region for 8X-Histidine tag is underlined
Saro2595-pVP_T51A_R	5’-CGCCGAGCGAATAGACCTGGAAAG-3’	underlined base does not match sequence in pVP302K/Ntag-2595
Saro2595-pVP_T51A_F	5’-CGCCCAACGGGCAGAAGG-3’	
Saro2595-pVP_Y166F_R	5’-GGCGAAGTTGTAGAAGTGGCCGAAG-3’	underlined base does not match sequence in pVP302K/Ntag-2595
Saro2595-pVP_Y166F_F	5’-CCGATCAAGATCGAGTACGCGATCG-3’	
Saro2595-pVP_Y224F_R	5’-CGAAGGCTTCGCCGCGGTAG-3’	underlined base does not match sequence in pVP302K/Ntag-2595
Saro2595-pVP_Y224F_F	5’-GCGAGGCGGCGACCTTCCTG-3’	
pVP302K-EcYghU F	5’-GATCGCAGGAATGACAGACAATACTTATCAGCCCGCGAAAG-3	underlined region matches sequence in pVP302K
pVP302K-EcYghU R	5’-CGGCTTTCTGTTACCCCTGACGCTTATCTTCCGTATTCGTC-3’	underlined region matches sequence in pVP302K
EcYghU-pVP302K F	5’-TCAGGGGTAACAGAAAGCCGAAAATAACAAAGTTAGCCTGAGCTG-3’	underlined region matches sequence in *yghU*
EcYghU-pVP302K R	5’-TGTCTGTCATTCCTGCGATCGCGCTCTGAAAATACAGATTTTCG-3’	underlined region matches sequence in *yghU*
pVP302K-HiFi-ATW-R	5’-TCCTGCGATCGCGCTCTGAAAATACAGATTTTCG-3’	
pVP302K-HiFi-ATW-F	5’-CAGAAAGCCGAAAATAACAAAGTTAGCCTGAGCTG-3’	
EcYfcG-pVP-Ntag-HiFi-F	5’-gtattttcagagcgcgatcgcaggaATGATCGATCTCTATTTCGCCCCGACAC -3’	lowercase region complementary to “pVP302K-HiFi-ATW-R”
EcYfcG-pVP-Ntag-HiFi-R	5’-ctaactttgttattttcggctttctgTTAACTATCCGAACGCTCATCACCGAGTTG -3’	lowercase region complementary to “pVP302K-HiFi-ATW-F”
SYK6 yghU pVP fix R	5’-gttattttcggctttctgttaagCTTCGGTCTTCG-3’	Lowercase region complementary to “SYK6 yghU pVP fix F”
SYK6 yghU pVP fix F	5’-cttaacagaaagccgaaaataacAAAGTTAGCCTGAG-3’	Lowercase region complementary to “SYK6 yghU pVP fix R”

Construction of *N. aromaticivorans* mutants.

Biological reagents. All PCR reactions were performed with Herculase II polymerase (Agilent Technologies, Santa Clara, CA). Primers were phosphorylated with polynucleotide kinase from Promega (Madison, WI). All other enzymes were from New England Biolabs (Ipswich, MA). All primers were from Integrated DNA Technologies (Coralville, IA).

Construction of pK18msB-MCS1. Plasmid pK18*mobsacB* (12) was amplified via PCR with phosphorylated primers "pK18msB AseI ampl F" and "pK18msB –MCS XbaI R". The product was circularized with T4 DNA ligase, then transformed into *E. coli* DH5α. The final 5278-base pair (bp) plasmid, pK18msB-MCS1, is similar to pK18*mobsacB*, except that the multiple cloning site has been removed, and a new XbaI site is 24 bp from one of the plasmid's native AseI sites (with the other native AseI site removed).

Plasmids for deleting Saro_1879 or Saro_2595. Regions of *N. aromaticivorans* genomic DNA containing either Saro_1879 or Saro_2595, along with ~1300-1600 bp flanking regions upstream and downstream of each gene, were separately amplified via PCR using the primer pairs "Saro1879 lvnsucr ampl F AseI" / "Saro1879 lvnsucr ampl R XbaI" and "Saro_2595 amp AseI for" / "Saro_2595 amp XbaI rev". The amplified DNA fragments were digested with AseI and XbaI, then ligated with T4 DNA ligase into AseI- and XbaI-digested pK18msB-MCS1. The resulting plasmids (pK18msB/Saro1879 and pK18msB/Saro2595) were transformed into *E. coli* (strain DH5α for the Saro_1879 plasmid and Turbo (New England Biolabs) for the Saro_2595 plasmid). PCR was performed on the purified plasmids using the phosphorylated primer pairs "Saro1879 lvnsucr del REV" / "Saro1879 lvnsucr del FOR" or "Saro_2595 del rev" / "Saro_2595 del for" to generate linear plasmids lacking the majority of the Saro_1879 and Saro_2595 coding regions, respectively (see Supplemental Fig. S10 for the Saro_2595 genomic region). These DNA fragments were circularized with T4 DNA ligase to form plasmids pK18msB/ΔSaro1879 and pK18msB/ΔSaro2595.

Deletion of Saro_1879. *N. aromaticivorans* has been reported to use sucrose as sole carbon source (6), but we found it to be incapable of growing in the presence of ≥10% sucrose (Supplemental Table S4). We noticed that the gene Saro_1879 is annotated as *sacB*, whose product, levansucrase, makes sucrose inhibitory to growth of many Gram-negative bacteria (15). To create an *N. aromaticivorans* strain whose genome we could modify using a *sacB*-containing plasmid, we deleted Saro_1879 from its genome. To do this, plasmid pK18msB/ΔSaro1879 was mobilized into *N. aromaticivorans* DSM 12444 via electroporation using a single 2.5 kV pulse in a 0.2 cm cuvette in a MicroPulser apparatus (Bio-Rad, Hercules, CA). *N. aromaticivorans* was made electrocompetent by washing exponential phase cells from LB cultures twice with ice-cold 0.5 M glucose, then resuspending the cells into 10% glycerol. Transformants in which the plasmid was integrated into the genome via homologous recombination (single-crossovers) were selected for by growth on solid LB containing kanamycin. These strains were grown in liquid LB to allow plasmid loss via homologous recombination, then plated on solid LB containing 10% sucrose to select for sucrose-tolerance. Sucrose-tolerant strains in which Saro_1879 had been deleted from the genome were confirmed by PCR amplification and sequencing of genomic DNA. One of these strains (12444Δ1879) was used as the parent strain for subsequent genetic modifications. 12444Δ1879 behaved the same as wild-type *N. aromaticivorans* DSM 12444 with respect to GGE metabolism.

Deletion of Saro_2595. To inactivate Saro_2595, we electroporated plasmid pK18msB/ΔSaro2595 into strain 12444Δ1879, and a strain lacking the majority of the Saro_2595 gene (referred to as 12444Δ2595; Supplemental Fig. S10B) was isolated via the process described above for deleting Saro_1879.

Replacement of Saro_2595 in the *N. aromaticivorans* genome with *E. coli yghU*. To generate a strain (referred to as 12444EcyghU) in which Saro_2595 was replaced in the *N. aromaticivorans* genome with the *E. coli yghU* gene (Supplemental Fig. S10C), we amplified the *yghU* gene from *E. coli* DH5α genomic DNA using primers "D2595pK18-EcYghU F" and "D2595pK18-EcYghU R", which each contain a sequence on their 5' end that is complementary to a region in the plasmid pK18msB/ΔSaro2595. pK18msB/ΔSaro2595 was amplified with the primers "EcYghU-D2595pK18 F" and "EcYghU-D2595pK18 R", which each contain a sequence on their 5' end that is complementary to *E. coli yghU*, to generate a linear fragment in which pK18msB-MCS1 contains the regions flanking Saro_2595 in the *N. aromaticivorans* genome, along with the regions complementary to *yghU*.

The PCR amplified fragments were connected using NEBuilder HiFi DNA Assembly Master Mix (New England Biolabs) (using 81 ng of the linear pK18msB/ΔSaro2595 fragment and 22 ng of the *yghU* fragment) and transformed into NEB 5-alpha competent *E. coli* (New England Biolabs). The resulting plasmid (pK18msB/EcyghU-Δ2595) consisted of pK18msB-MCS1 containing the *E. coli yghU* gene flanked by the regions that flank Saro_2595 in the *N. aromaticivorans* genome (i.e. with the start and stop codons positioned where the respective codons of Saro_2595 would naturally be; Supplemental Fig. S10C).

This plasmid was mobilized into 12444Δ2595 via conjugation from *E. coli* S17-1. Transconjugants (single-crossovers) of *N. aromaticivorans* were isolated on solid GluSis containing kanamycin. After growth in liquid GluSis, strains that lost the plasmid and retained the *yghU* gene at the native Saro_2595 locus were isolated on solid GluSis medium containing sucrose. The presence of the *yghU* gene in the genome was confirmed via PCR and sequencing.

Figure S10: Genotypes of the Saro_2595 locus in *Novosphingobium aromaticivorans* strains used in this study. The coding region for Saro_2595 is shown in orange, and the coding region for the *E. coli yghU* gene is in blue. Numbers in the displays are the genomic DNA position numbers in the NCBI entry for the *N. aromaticivorans* genomic sequence. Where present, the start and stop codons for genes are shown. (A) The Saro_2595 locus in strain 12444Δ1879 (the wild-type version of the locus). (B) The Saro_2595 locus in strain 12444Δ2595. In this strain, the majority of the Saro_2595 coding sequence has been removed from the genome, along with the two base pairs upstream of the gene's translational start codon. The last 34 base pairs of the coding region remain in the genome. (C) The Saro_2595 locus in strain 12444EcyghU. The *yghU* gene from *E. coli* DH5α was placed into the genome so that the start and stop codons were in the same positions as those of the native Saro_2595.

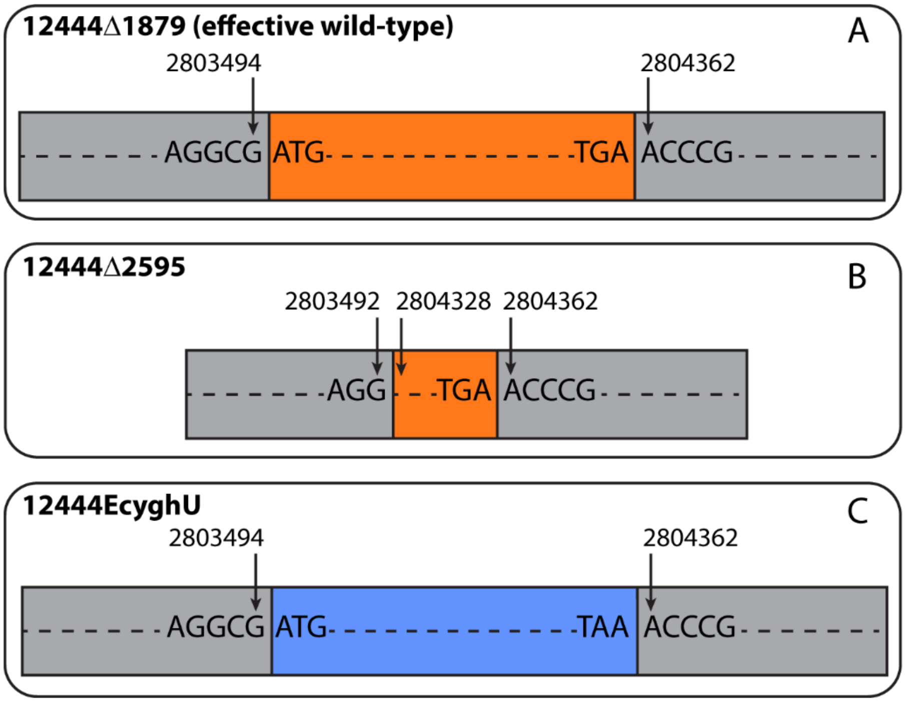

Table S4: Relationship between Klett Units (KU) and colony forming units (CFUs) for *Novosphingobium aromaticivorans* +/- sucrose (CFU mL^{-1} KU^{-1})

	-sucrose	+sucrose
DSM 12444	8.0 (± 3.2) × 10^6	0
12444Δ1879	8.1 (± 1.7) × 10^6	7.3 (± 3.4) × 10^6

Cultures of *Novosphingobium aromaticivorans* DSM 12444 and 12444Δ1879 were grown in liquid medium, and cell densities were measured using a Klett-Summerson photoelectric colorimeter with a red filter. Cultures were then diluted, and dilutions were plated onto solid media +/- 10% sucrose.

Expression and purification of recombinant proteins

Plasmid for expressing recombinant NaGST$_{Nu}$. Saro_2595 was amplified from *N. aromaticivorans* genomic DNA with the primers "Saro2595 Ctag PciI F" and "Saro2595 Ctag BsaI R". This fragment was digested with PciI and BsaI. The expression vector pVP302K (13) was amplified using the primers "pVP302K Ctag BsaI F" and "pVP302K Ctag NcoI R". This fragment was digested with BsaI and NcoI. The digested fragments were ligated using T4 DNA ligase, generating plasmid pVP302K/Ctag-2595, which consists of a T5 promoter followed by the coding sequences of Saro_2595, the RtxA protease from *Vibrio cholerae*, and a His$_8$ tag.

pVP302K/Ctag-2595 was amplified using phosphorylated primers "Ctag 2595-pVP add Stop R" and "pVP302K Ntag HindIII F". This fragment was circularized using T4 DNA ligase to generate plasmid pVP302K/Untagged2595, in which a stop codon has been introduced directly after Saro_2595. pVP302K/Untagged2595 was amplified via PCR using phosphorylated primers "2595-pVP C to Ntag F" and "pVP302 C to Ntag R". This fragment was circularized using T4 DNA ligase to generate plasmid pVP302K/Ntag-2595, which contains a T5 promoter followed by coding sequences for a His$_8$-tag, a tobacco etch virus (TEV) protease recognition site and Saro_2595.

Plasmids for expressing recombinant NaGST$_{Nu}$ variants. Plasmids were constructed to express versions of NaGST$_{Nu}$ with the following single amino acid modifications: Threonine 51 into Alanine (T51A), Tyrosine 166 into Phenylalanine (Y166F), and Tyrosine 224 into Phenylalanine (Y224F). pVP302K/Ntag-2595 was amplified by the following three sets of phosphorylated primers: "Saro2595-pVP_T51A_R" and "Saro2595-pVP_T51A_F"; "Saro2595-pVP_Y166F_R" and "Saro2595-pVP_Y166F_F"; and "Saro2595-pVP_Y224F_R" and "Saro2595-pVP_Y224F_F". The three resulting linear DNA fragments were individually circularized using T4 DNA ligase, then transformed into *E. coli* DH5α. The resulting plasmids, pVP302K/Ntag-2595-T51A, pVP302K/Ntag-2595-Y166F, and pVP302K/Ntag-2595-Y224F, were purified, then used for expression of NaGST$_{Nu}$ variants T51A, Y166F, Y224F, respectively.

Plasmid for expressing recombinant *E. coli* YghU. The *yghU* gene was amplified from *E. coli* DH5α genomic DNA using the primers "pVP302K-EcYghU F" and "pVP302K-EcYghU R". pVP302K was amplified by PCR using the primers "EcYghU-pVP302K F" and "EcYghU-pVP302K R". The two amplified fragments, with ends that are complementary to each other, were concurrently transformed (94 ng linear pVP302K, 168 ng *yghU* gene, in 4 µL TE buffer) into *E. cloni* 10G chemically competent cells (Lucigen, Middleton, WI). The fragments were combined via homologous recombination *in vivo* (16), and the resulting plasmid, pVP302K/Ntag-ecyghU, was purified from the cells and verified via sequencing.

Plasmid for expressing recombinant *E. coli* YfcG. The *yfcG* gene was amplified from *E. coli* DH5α genomic DNA using the primers "EcYfcG-pVP-Ntag-HiFi-F" and "EcYfcG-pVP-Ntag-HiFi-R". pVP302K was amplified via PCR using the primers "pVP302K-HiFi-ATW-R" and "pVP302K-HiFi-ATW-F". The two amplified fragments, with ends that are complementary to each other, were combined using NEBuilder HiFi DNA Assembly Master Mix (New England Biolabs) (100 ng linear pVP302K, 48 ng yghU gene) and transformed into NEB 5-alpha competent *E. coli* (New England Biolabs). The resulting plasmid, pVP302K/Ntag-ecyfcG, was purified and verified via sequencing.

Plasmid for expressing recombinant SYK6GST$_{Nu}$. A fragment containing the SLG_04120 gene, codon-optimized for *E. coli*, with ends complementary to pVP302K, was ordered as a gBlock from New England Biolabs. The sequence of the fragment was:

5’-GTATTTTCAGAGCGCGATCGCAGGAATGGCCGACTCAGATCCATCCATGAATCAGCCGAC
GGGTTACGTCCCGCCGAAAGTTTGGACCTGGGACAAAGAGAACGGCGGTCAGTTCAGCAAT
ATCAACGCCCCTACGGCTGGTGCGCGCCAGGACGTCACGCTCCCTGTAGGGGAGCACCCTAT
CCAATTATATAGTCTCGGCACTCCGAATGGTCAGAAAGTTACTATCATGTTGGAAGAACTGC
TGGCTGCTGGCTTTGATGCTGAGTATGACGCCTGGCTCATCAAAATCTACACAGGCGAGCAA
TTCGGATCTGATTTCGTCGCCATTAACCCTAATAGCAAAATTCCGGCTATGATGGACCATGG
TCTCGATCCGCCGCTCCGTTTATTTGAGTCTGGTTCTATGTTAGTTTATCTGGCCGAAAAGTT
TGGCGCATTCCTCCCGACCGAAATCCGCAAACGTACGGAAACCTTTAACTGGCTCATGTGGC
AGATGGGTTCTGCTCCTTTTGTGGGTGGTGGCTTTGGCCACTTCTATGCGTACGCCCCATTTA
AAATCGAATATGCCATTGATCGTTACGCGATGGAAACCAAGCGCCAACTGGACGTTCTGGAT
AAAAATCTGGCCGATCGTGAATTTATGATCGGCGATGAAATCACCATCGCAGATTTTGCGAT
TTTCCCTTGGTACGGCTCGATTATGCGTGGCGGTTACAACGCGCAAGAATTCTTGAGCACTC
ACGAGTACCGTAACGTTGATCGCTGGGTTACGCAGCTTTCTGAACGTACGGGCGTAAAGCGT
GGTCTCCTTGTCAATTCCGCGGGTCGCCCGGGAGGTGGCATTGCGGAACGCCATAGCGCGGC
TGATTTAGACGCGTCGATTAAAGCGGCTGAACAAGAGGCCGCGAAGACCGAAGCTtAACAG
AAAGCCGAAAATAACAAAGTTAG-3’
(underlined regions match sequences in pVP302K)

pVP302K was amplified via PCR using the primers “pVP302K-HiFi-ATW-R” and “pVP302K-HiFi-ATW-F”. The resulting linear pVP302K fragment and the SLG_04120 gBlock were concurrently transformed (57 ng linear pVP302K and 84 ng SLG_04120, in 10 µL) into *E. cloni* 10G chemically competent cells (Lucigen, Middleton, WI). The fragments were combined via homologous recombination *in vivo* (16). Sequencing revealed that a single base pair was missing from the SLG_04120 gene sequence (the lowercase t near the end of the above sequence) in the resulting plasmid. The plasmid was thus amplified using the primers “SYK6 yghU pVP fix R” and “SYK6 yghU pVP fix F” to generate a linear fragment with ends complementary to each other containing the correct sequence of the gene. This fragment was transformed into *E. cloni* 10G chemically competent cells, and its ends were combined *in vivo* via homologous recombination. The circularized plasmid, pVP302K/Ntag-SLG_04120, was purified from these cells and verified via sequencing.

Purification of recombinant proteins. Recombinant proteins were purified as described previously (17), except that cells were lysed by sonication, first using a Branson Sonifier 450 (Branson Ultrasonics, Danbury, CT) (duty cycle 50%, output level 6, for six rounds of 1-2 min), then a Qsonica Q500 with a cup horn attachment (Qsonica, Newtown, CT) (60% amplitude for 10 minutes with cycles of 10 s on, 10 s off). His_8-tagged proteins were purified from lysates over a column packed with Ni^{2+}-NTA resin (Qiagen, Hilden, Germany) attached to an AKTAprime plus FPLC (GE Healthcare Life Sciences), then incubated with TEV protease to remove the His_8-tag. The protease reaction mixture was passed through a Ni^{2+}-NTA column to separate the recombinant protein from the cleaved His_8-tag and TEV protease (which also contained a His_8-tag).

Identification and quantification of extracellular metabolites

Initial identification using LC-MS

Compounds were separated on a Phenomenex PFP 250 x 4.6mm column attached to an Accela LC pump equipped with a PDA UV detector. Running buffers A (5 mM formic acid and 5% acetonitrile in H_2O) and B (methanol) were initially at 82.5% and 17.5%, respectively. Buffer B was held at 17.5% for 18 minutes, then increased to 50% over 5 minutes, held at 50% for 3 minutes, then returned to initial conditions and re-equilibrated for 4 minutes. Flow rate was 1 mL per minute. UV absorbance data from 190 - 500 nm at 5 Hz and single wavelength data at 254 nm (20 Hz, 9 nm bandwidth) were collected.

Samples were analyzed by high resolution, tandem mass spectrometry using a Thermo Scientific Q Exactive Orbitrap mass spectrometer. The mass spectrometer was operated in fast polarity switching mode with acquisition of MS/MS spectra of the two most abundant precursor ions from the preceding MS1 scan (50-750 Th). Resolution was 35,000 at 200 Th for MS1 scans and 17,500 at 200 Th for MS/MS scans. Capillary voltage was set at 4000V in both polarities, sheath gas at 50 units, auxiliary gas at 20 units, probe heater at 350°C, inlet capillary at 325°C, and the S-Lens at 50 units. AGC target was 1e6 for MS1 scans and 2e5 for MS/MS scans with a maximum injection time of 50 ms. The isolation width for MS/MS scans was set to 2 Th and a 5 s dynamic exclusion time was used.

Elemental compositions of the metabolites were derived from the mass measurements. From the MS/MS fragmentation patterns and previous data, we provisionally identified the metabolites. We used standards to confirm these putative identifications by matching retention times and mass spectra.

Figure S11: After metabolites were identified using LC-MS (see above), routine HPLC analysis was performed using an Ultra AQ C18 5 µm column attached to a System Gold HPLC (see main text) running either a 34 min or 43 min method. (A) Compositions of the running buffer in the HPLC methods used. Samples from growth experiments were typically run using the 43 min method (black line), and samples from *in vitro* enzyme assays were typically run using the 34 min method (orange line). Percent methanol in the running buffer is shown. The remainder of the running buffer was Buffer A (5 mM formic acid, 5% acetonitrile in H_2O), and the flow rate was 1 mL/minute. (B) Absorbance (280 nm) and retention times of metabolites analyzed using the 43 min method. Retention times of metabolites analyzed using the 34 min method are shown in Supplemental Fig. S4.

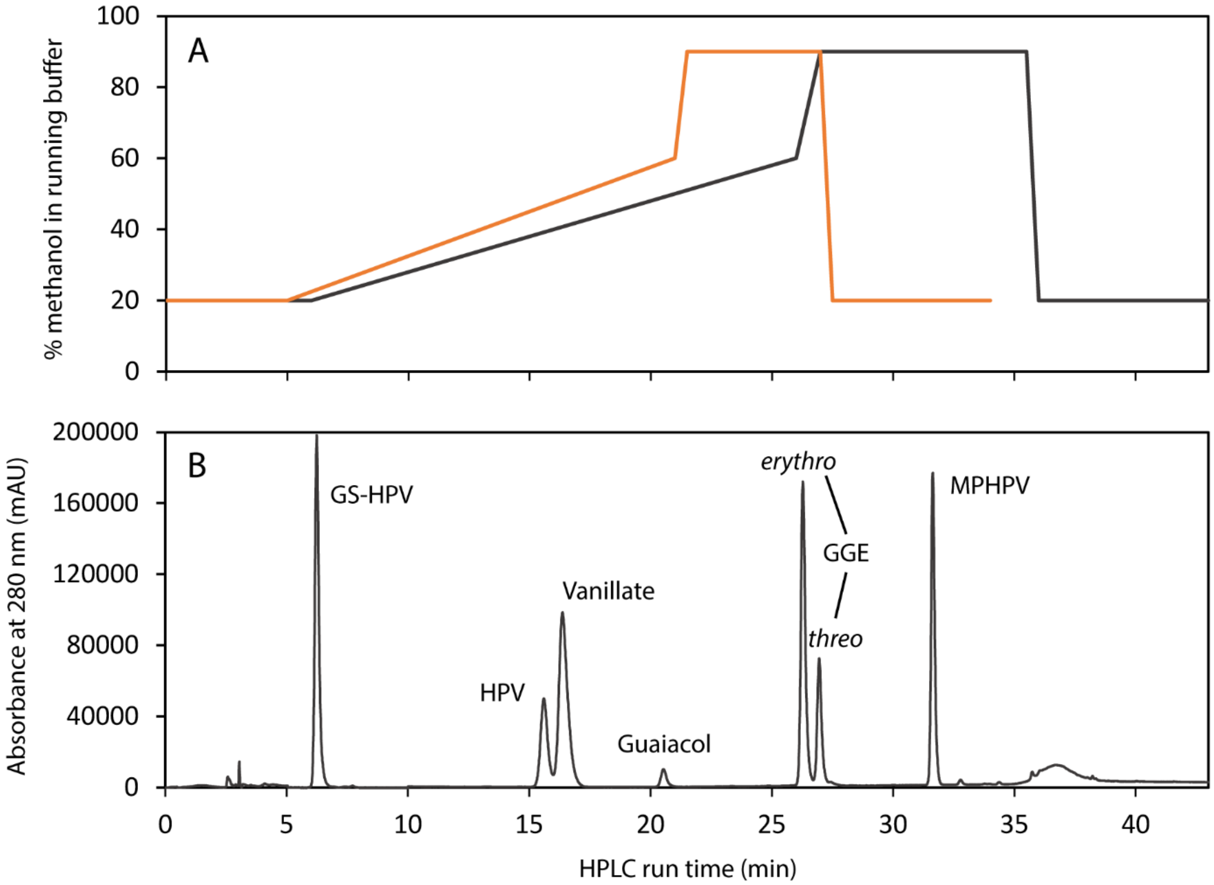

Table S5: Primers used for RT qPCR of *Novosphingobium aromaticivorans* genes.

Transcript assayed for	Primers
Saro_0141 (*rpoZ*)	5'-GAGATCGCGGAAGAAACCGTGC-3'<br>5'-GATTTCATCCACCTCGTCGTCGTC-3'
Saro_0205 (*ligD*)	5'-CAACATCAAGTCGAACATCGCGGAAG-3'<br>5'-CTGGTGGATCGAATGCAGCGAG-3'
Saro_0793 (*ligO*)	5'-GATCGAGGAATCTTCCTACGACGACTG-3'<br>5'-GTTTACCACGCCGTGGAGGTTCAC-3'
Saro_0794 (*ligN*)	5'-CATATCGTCTGCACCGCTTCGATGTC-3'<br>5'-GCAGAATGCCGAGCAGATCACG-3'
Saro_1875 (*ligL*)	5'-CCATGTCGTCAACACCGCATCG-3'<br>5'-CATGTTCTCGGTCAGGTTCAGCAC-3'
Saro_2091 (*ligF*)	5'-GCTGCTGACGGTGTTCGAGAAG-3'<br>5'-CTTGAACCAGTCGGTGTGATGCTC-3'
Saro_2405 (*ligP*)	5'-CATCGTCGAATACCTCGATGCCAAGTATC-3'<br>5'-GTCCTGGCAGAAGCAGAACATCCAC-3'
Saro_2595	5'- CCACGATCATGCTGGAAGAACTGCTC-3'<br>5'-GATTCGAAGACGCGGAACGGTTCAG-3'

SUPPORTING INFORMATION REFERENCES

1. Stamatakis, A. (2014) RAxML version 8: a tool for phylogenetic analysis and post-analysis of large phylogenies. *Bioinformatics* 30(9), 1312–1313.
2. Ohta, Y., Nishi, S., Hasegawa, R., Hatada, Y. (2015) Combination of six enzymes of a marine *Novosphingobium* converts the stereoisomers of β-O-4 lignin model dimers into the respective monomers. *Sci Rep* 5, 15105.
3. Fredrickson, J.K., Brockman, F.J., Workman, D.J., Li, S.W., Stevens, T.O. (1991) Isolation and characterization of a subsurface bacterium capable of growth on toluene, naphthalene, and other aromatic compounds. *Appl Environ Microbiol* 57(3), 796–803.
4. Fredrickson, J.K., et al. (1995) Aromatic-degrading *Sphingomonas* isolates from the deep subsurface. *Appl Environ Microbiol* 61(5), 1917–1922.
5. Notomista, E., et al. (2011) The marine isolate *Novosphingobium* sp. PP1Y shows specific adaptation to use the aromatic fraction of fuels as the sole carbon and energy source. *Microb Ecol* 61(3), 582–594.
6. Stolz, A., et al. (2000) Description of *Sphingomonas xenophaga* sp. nov. for strains BN6T and N,N which degrade xenobiotic aromatic compounds. *Int J Syst Evol Microbiol* 50 Pt 1, 35–41.
7. Pal, R., Bhasin, V.K., Lal, R. (2006) Proposal to reclassify [*Sphingomonas*] *xenophaga* Stolz et al. 2000 and [*Sphingomonas*] *taejonensis* Lee et al. 2001 as *Sphingobium xenophagum* comb. nov. and *Sphingopyxis taejonensis* comb. nov., respectively. *Int J Syst Evol Microbiol* 56(Pt 3), 667–670.
8. Taylor, R.G., Walker, D.C., McInnes, R.R. (1993) *E. coli* host strains significantly affect the quality of small scale plasmid DNA preparations used for sequencing. *Nucleic Acids Res* 21(7), 1677–1678.
9. Simon, R., Priefer, U., Pühler, A. (1983) A Broad Host Range Mobilization System for In Vivo Genetic Engineering: Transposon Mutagenesis in Gram Negative Bacteria. *Nat Biotech* 1(9), 784–791.
10. Wood, W.B. (1966) Host specificity of DNA produced by *Escherichia coli*: bacterial mutations affecting the restriction and modification of DNA. *J Mol Biol* 16(1), 118–133.
11. Doherty, A.J., Ashford, S.R., Brannigan, J.A., Wigley, D.B. (1995) A superior host strain for the over-expression of cloned genes using the T7 promoter based vectors. *Nucleic Acids Res* 23(11), 2074–2075.
12. Schäfer, A., et al. (1994) Small mobilizable multi-purpose cloning vectors derived from the *Escherichia coli* plasmids pK18 and pK19: selection of defined deletions in the chromosome of *Corynebacterium glutamicum*. *Gene* 145(1), 69–73.
13. Gall, D.L., Ralph, J., Donohue, T.J., Noguera, D.R. (2014) A group of sequence-related sphingomonad enzymes catalyzes cleavage of β-aryl ether linkages in lignin β-guaiacyl and β-syringyl ether dimers. *Environ Sci Technol* 48(20), 12454–12463.
14. Cohen-Bazire, G., Sistrom, W.R., Stanier, R.Y. (1957) Kinetic studies of pigment synthesis by non-sulfur purple bacteria. *J Cell Comp Physiol* 49(1), 25–68.
15. Gay, P., Le Coq, D., Steinmetz, M., Berkelman, T., Kado, C.I. (1985) Positive selection procedure for entrapment of insertion sequence elements in gram-negative bacteria. *J Bacteriol* 164(2), 918–921.
16. Bubeck, P., Winkler, M., Bautsch, W. (1993) Rapid cloning by homologous recombination in vivo. *Nucleic Acids Res* 21(15), 3601–3602.
17. Gall, D.L., et al. (2014) Stereochemical features of glutathione-dependent enzymes in the *Sphingobium* sp. strain SYK-6 β-aryl etherase pathway. *J Biol Chem* 289(12), 8656–8667.
